# Caffeine—Legal Natural Stimulant with Open Research Perspective: Spectroscopic and Theoretical Characterization

**DOI:** 10.3390/molecules29184382

**Published:** 2024-09-14

**Authors:** Teobald Kupka, Natalina Makieieva, Michał Jewgiński, Magdalena Witek, Barbara Blicharska, Oimahmad Rahmonov, Karel Doležal, Tomáš Pospíšil

**Affiliations:** 1 Faculty of Chemistry and Pharmacy, University of Opole, 48, Oleska Str., 45-052 Opole, Poland; 2 Department of Bioorganic Chemistry, Faculty of Chemistry, Wrocław University of Science and Technology, 27, Wybrzeże Wyspiańskiego Str., 50-370 Wrocław, Poland; michal.jewginski@pwr.edu.pl; 3 Department of Biotechnology and General Technology of Food, Faculty of Food Technology, University of Agriculture in Krakow, 122, Balicka Str., 30-149 Kraków, Poland; magdalena.witek@urk.edu.pl; 4 Faculty of Physics, Astronomy and Applied Computer Science, 11, Prof. Stanisława Łojasiewicza Str., 30-348 Kraków, Poland; b.blicharska@gmail.com; 5 Institute of Earth Sciences, Faculty of Natural Sciences, University of Silesia in Katowice, 41-200 Sosnowiec, Poland; oimahmad.rahmonov@us.edu.pl; 6 Laboratory of Growth Regulators, Institute of Experimental Botany AS CR & Faculty of Science, Palacký University, Šlechtitelů 27, 78371 Olomouc, Czech Republic; karel.dolezal@upol.cz (K.D.); tomas.pospisil@upol.cz (T.P.); 7 Department of Chemical Biology, Faculty of Science, Palacký University, Šlechtitelů 27, 78371 Olomouc, Czech Republic

**Keywords:** caffeine, ^17^O NMR, molecular structure, DFT, molecular modeling

## Abstract

Caffeine is an alkaloid with a purine structure and has been well known for centuries due to its presence in popular drinks—tea and coffee. However, the structural and spectroscopic parameters of this compound, as well as its chemical and biological activities, are still not fully known. In this study, for the first time, we report on the measured oxygen-17 NMR spectra of this stimulant. To support the assignment of our experimental NMR data, extensive quantum chemical calculations of NMR parameters, including nuclear magnetic shielding constants and indirect spin–spin coupling constants, were performed. In a theoretical study, using nine efficient density functionals (B3LYP, BLYP, BP86, CAM-B3LYP, LC-BLYP, M06, PBE0, TPSSh, wB97x), and in combination with a large and flexible correlation-consistent aug-cc-pVTZ basis set, the structure and NMR parameters were predicted for a free molecule of caffeine and in chloroform, DMSO and water. A polarized continuum model (PCM) was used to include a solvent effect. As a result, an optimal methodology was developed for predicting reliable NMR data, suitable for studies of known, as well as newly discovered, purines and similar alkaloids. The results of the current work could be used in future basic and applied studies, including NMR identification and intermolecular interactions of caffeine in various raw materials, like plants and food, as well as in the structural and spectroscopic characterization of new compounds with similar structures.

## 1. Introduction

**Figure 1:**
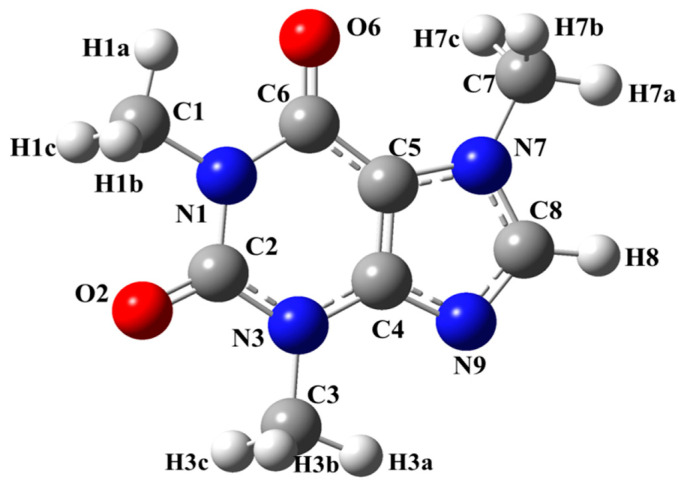
Caffeine structure with atom labeling.

Caffeine [1] (1,3,7-trimethyl-3,4,5,7-tetrahydro-1H-purine-2,6-dione) is one of the most popular neurostimulants in the world, and together with theophylline and theobromine, it belongs to methyl-xanthines. Its stimulating and hallucinogenic properties have been known to humanity since ancient times. From a chemical point of view, it is a purine alkaloid [1], which is most often consumed as a component of tea and coffee [2,3]. Its molecular structure in the gas phase has been reported on [1], and its chemical formula with atom labeling is shown in Figure 1.

Recently, caffeine could be purchased in the form of concentrated solutions—so-called “energy drinks”—as well as tablets. This substance is routinely used in medicine as a hypervascular agent [2]. In everyday life, caffeine is often consumed by schoolchildren, students and athletes to increase intellectual and physical performance. In addition, caffeine use plays a significant role in the work activities of some iconic representatives of science and art. Mathematician Paul Erdős attributed caffeine as a key factor in his working effectiveness, and after a 30-day pause in caffeine consumption, he had “trouble doing mathematics without coffee”, claiming that his “mathematical notes became blank pieces of paper” that he just “stared at, unable to work”. Another “connoisseur” of caffeine was the mathematician Alfred Renyi, the author of the aphorism “A mathematician is a machine for turning coffee into theorems”. It is also known that a famous painter W. H. Auden considered coffee to be one of the “labor-saving utensils” of his “intellectual kitchen”. Following his creative ambitions and struggling with frequent debts, the literary icon Honore de Balzac also drank large quantities (up to 50 cups per day) of strong coffee [4,5]. However, caffeine abuse does not lead to significant health problems, as in the case of cocaine or amphetamines or even heroin or morphine [2]. Moreover, world and government health organizations have not registered cases of drug addiction caused by caffeine. For this reason, there are no restrictions on the production of caffeine-containing products and no exact standards for the content of this alkaloid in these products [2]. However, some scientific sources demonstrate that caffeine abuse leads to negative, although reversible, consequences for human health. Concentration-dependent increases in blood pressure, urination frequency and anxiety were recorded [2,3]. In the case of people with diseases of the cardiovascular and urinary systems, these facts could not be neglected. Moreover, people with genetic predisposition and other factors that increase the risk of heart, vascular and kidney diseases should also pay special attention to the frequency and amount of caffeine consumption. For this reason, it is extremely important to develop methods that allow for the identification and quantification of the amount of caffeine contained in humans’ consumed products. The literature provides data on the use of various chromatographic and spectroscopic methods for this purpose—high-performance liquid chromatography (HPLC) with ultraviolet detection (UV) [6,7], mass spectrometry (MS) [8,9,10], particle beam/electron ionization mass spectrometry (PB/EIMS) [11], Fourier transform near-infrared reflectance (FT-NIR) spectroscopy [12] and nuclear magnetic resonance (NMR) spectroscopy [13,14,15].

As can be noticed, the history of experimental and theoretical studies on caffeine is quite diverse and extensive [1,3,9,10], but this stimulant is still open to newly designed chemical studies. One of the relatively new methods for the characterization of natural products’ properties is the in silico prediction of some of their parameters. Below, we will mention a few recent theoretical studies on the description of some structural, spectroscopic [16] and chemical reactivity parameters on a free molecule of caffeine and in solution, using an approximate solvent effect only via a polarizable continuum model (PCM [17,18]). Gibson and Fowler [19] studied the aromaticity of methyl-xanthines, including caffeine at low levels of theory (HF/6-31G* and B3LYP/6-31G*). They revealed a strong delocalized π-electron current above the imidazole ring of caffeine molecules. Another density functional theory (DFT [20,21]) study by Rijal et al. [22] concentrated on nicotine and caffeine in the gas phase and solution employing B3LYP/6-311++G**. They discussed dipole moment changes in free molecules and the magnitude of changes as a result of solvent impacts. Additionally, they calculated global reactivity descriptors, density of states (DOSs), atomic charges and vibrational and electronic spectra. Gomez et al. [23] reported on a DFT analysis of conformational preferences in caffeine, employing B3LYPD3/6-311++G** calculations with the single-point domain-based local pair natural orbital coupled cluster [24] (DLPNO-CCSD(T)) refinement of energy at stationary points. They concluded that there was the presence of a nearly free rotation or fluctuation in the methyl groups in caffeine.

As can be seen from Figure 1, caffeine contains hydrogen, carbon, nitrogen and oxygen elements in its structure. However, only experimental ^1^H, ^13^C and ^15^N NMR spectra [25] have been reported. Thus, to our surprise, no ^17^O NMR spectra of caffeine are available in the literature [25].

Several original experimental studies and review papers on the application of oxygen-17 NMR to characterize organic and inorganic molecules have been reported [26,27,28,29,30,31,32,33,34,35,36]. Additionally, theoretical predictions of ^17^O NMR have been also reported [37,38,39]. Following Krivdin’s review, it is worth citing well-defined difficulties in running 17O NMR spectra: “The ^17^O NMR signals are difficult to detect even for small inorganic and organic molecules. Furthermore, ^17^O isotope has a very low natural abundance of only 0.038%, so that it is routinely observable only for neat liquids and solutions of very high concentrations or, alternatively, in the ^17^O-enriched samples.” Moreover, among the known deficiencies of ^17^O nuclei is the very low natural receptivity in comparison to protons (1.11 × 10^−5^). Its spin of 5/2 leads to very broad lines, in particular in asymmetric environments. Low Larmor frequency results in acoustic ringing (rolling baseline). Therefore, highly concentrated solutions could be studied. Taking the above into account, the lack of natural abundance oxygen-17 NMR reports could be due to the difficulties of experimental studies of this isotope by using the NMR technique.

On the other hand, Colherinhas et al. [40] recently reported on DFT calculated chemical shifts in caffeine in water using four density functionals (B3LYP, CAM-B3LYP, BHandHLYP and PBE1PBE) combined with a relatively small Pople-type 6-311++G(2d,2p) basis set. The authors reported on the B3LYP/aug-cc-pVDZ geometry optimization of caffeine in water using the PCM approach for the subsequent gauge-including atomic orbital (GIAO [41,42]) NMR calculation of nuclear magnetic shielding tensors. Interestingly, Colherinhas et al. [40] used experimental data from Ulrich et al. [43] for comparison with their predicted values. However, they did not report [40] experimental chemical shifts in three different methyl carbons in caffeine molecules. Obviously, in our opinion, the authors [40] could compare their theoretical chemical shifts to already-reported proton and carbon NMR data in chloroform [25]. Additionally, in comparison to a more complete and flexible triple-zeta aug-cc-pVTZ Dunning-type basis set, they used [40] a significantly smaller aug-cc-pVDZ one. This basis set is known to be very unreliable and prone to producing large errors [44,45].

For this reason, we decided to expand the existing knowledge about the spectroscopic properties of caffeine and present in this work its first experimental ^17^O NMR spectrum. In addition, the current development of analytical techniques makes it possible to discover more and more structurally similar plant metabolites. Quests for new substances, originating from both new and long-known plant species, are popular in the literature [13]. In this case, molecular modeling methods are frequently used to support the determination of bioactive metabolite structure and interpret the spectroscopic properties of novel compounds. For this reason, in the current study, we are aiming at developing an efficient theoretical methodology for predicting reliable NMR properties of caffeine and similar alkaloids. Therefore, we tested nine density functionals, including B3LYP, BLYP, BP86, CAM-B3LYP, LC-BLYP, M06, PBE0, TPSSh and wB97X, and applied them to study the NMR properties of caffeine in vacuum and three solvents (chloroform, DMSO and water). To verify the accuracy of the calculated results, the theoretical predicted parameters were compared with reported ^1^H, ^13^C and ^15^N NMR data [25], as well as with our experimental results for ^17^O NMR.

## 2. Materials and Methods

### 2.1. Materials

A caffeine sample was purchased from Fagron Ltd. (Krakow, Poland). Its purity was checked with ^1^H NMR spectra in D_2_O (only a residual HOD peak was visible, and there were no other signals, indicating a lack of any impurities). Chloroform-d from Aldrich was used for ^17^O NMR spectra.

### 2.2. NMR Measurements

The only magnetic active oxygen nucleus is ^17^O with a spin of 5/2, natural abundance of 0.038% and natural receptivity relative to ^13^C of 6.50 × 10^−2^ [26,27,28,29]. These parameters indicate broad signals present in ^17^O NMR spectra and very low sensitivity. Luckily, the relaxation times are short, making the fast accumulation of spectra feasible. Initially, the standard ^1^H NMR spectra of caffeine in CDCl_3_ were measured at room temperature using a Jeol ECZ400R (400 MHz, 9.39 Tesla) NMR spectrometer equipped with a Royal HFW probe. Next, the ^17^O NMR spectra were recorded at 54.196 MHz in CDCl_3_ at 35 °C for increased solubility. A spectral width of 500 ppm and offset of 200 ppm were selected. Oxygen-17 spectra were referenced to an external standard (H_2_O), and the following parameters were used: 1,048,576 scans, 8192 data points, 0.2 s relaxation delay, 13.95 μs pulse width (90°) and repetition time 0.44117 s. Finally, 100 Hz line broadening was applied in the post-processing procedure.

### 2.3. Theoretical Methods

All calculations were performed with the Gaussian 16 program package [46,47]. For convenience, instead of presenting the names of all density functionals in figures, they were labeled with the following consecutive numbers: B3LYP [48,49,50] (1), BLYP [51] (2), BP86 [48] (3), CAM-B3LYP [52] (4), LC-BLYP [53] (5), M06 [54] (6), PBE0 [55] (7), TPSSh [56] (8) and wB97X [57] (9). In the first step, the structure of an isolated caffeine molecule was fully optimized with the selected density functionals, combined with the aug-cc-pVTZ basis set. The molecular structure of caffeine in the gas phase was reported on [1]. Unconstrained geometry optimizations were conducted with very tight convergence criteria in vacuum and in the presence of chloroform, DMSO and water using the polarized continuum model (PCM) [58]. The optimized structural parameters closely reproduced the experimental structure of caffeine in the gas phase [1]. For brevity, all the structures optimized with the selected functionals in different solvents are included in the Supplementary Materials (Appendix I–IX). The abovementioned density functionals were shown in the literature as being efficient in the prediction of nuclear magnetic shieldings, chemical shifts and indirect spin–spin coupling constants (SSCCs) for different classes of alkaloids [59]. The lack of imaginary frequencies confirmed the equilibrium (ground state) structure of caffeine in different environments. The prediction of NMR parameters was performed at the same level of theory as for optimization. Nuclear shieldings were calculated with the GIAO approach [41,42]. ^1^H, ^13^C, ^15^N and ^17^O chemical shifts were predicted using small molecules as theoretical references: TMS for carbon and protons, nitromethane for nitrogen and water for oxygen signals [60,61]. The theoretical chemical shift δ (i) for different density functionals and solutions was calculated as the difference between shieldings of reference σ (ref) and nuclei under question σ (i) using Equation (1):

δ(i) = σ(ref) − σ(i), in ppm(1)

Liquid water is used as a reference in experimental O-17 NMR studies, and it is necessary to include the gas-to-liquid shift for ^17^O of −36.1 ppm (taken from [62]). The ^n^J_C-H_, ^n^J_H-H_ and ^n^J_N-H_ coupling constants were calculated using the “mixed” basis set option [60]. 

## 3. Results

This paper is organized as follows. The experimental ^17^O NMR spectrum of caffeine in water is presented first, and next, the predicted theoretical ^1^H, ^13^C, ^15^N and ^17^O NMR spectra are discussed and compared with available experimental data.

### 3.1. ^17^O NMR Spectrum of Caffeine in CDCl3

**Figure 2:**
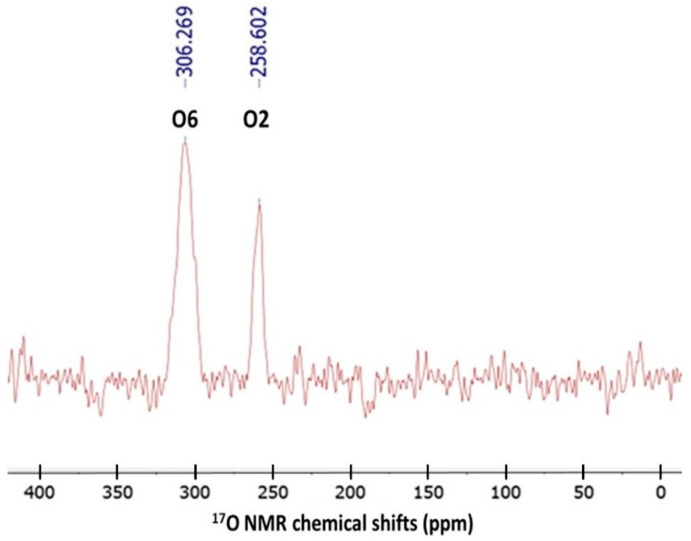
^17^O NMR spectrum of caffeine in CDCl_3_ measured at 35 °C (100 Hz line broadening).

The ^17^O NMR spectrum of caffeine in deuterochloroform is shown in Figure 2. Two broad and well-separated signals (ν_1/2_ = 50 Hz) are clearly seen at about 258.60 and 306.27 ppm. As expected, for low-frequency nuclei, a problem with very low sensitivity was noticed (over 1,000,000 scans at 35 °C were necessary). In addition, acoustic ringings were present as a rolling baseline, subsequently corrected in a polynomial fit and subtracted for the initial background. The spectral patterns seen in Figure 2 clearly indicate the presence of oxygen atoms in two different chemical environments. According to theoretical predictions discussed later, the signals were assigned to magnetically non-equivalent O2 and O6 atoms. In the subsequent part of this work, we will analyze the ^17^O NMR parameters of caffeine in more detail.

### 3.2. Theoretical Nuclear Magnetic Shielding Parameters of Caffeine

To avoid the overcrowding of the main text, most of the calculated NMR results were moved to the Supplementary Information, but some results will be discussed in the subsequent parts of this manuscript. The isotropic part of nuclear magnetic shielding tensors for caffeine nuclei was selected from GIAO NMR calculations. For brevity, the results obtained with nine density functionals in four environments, including the gas phase, chloroform, DMSO and water, were gathered in Table S1 in the Supplementary Materials. In the case of methyl groups, the ^1^H shieldings were averaged. It is apparent from Table S1 that some nuclei are insensitive to environment polarity. Thus, the B3LYP calculated C1 shielding in the gas phase and water are generally the same (156.38 vs. 156.26 ppm). C8 is more susceptible to the environment, and the following shieldings in the gas phase, CHCl_3_ and H_2_O are calculated: 40.04, 37.51 and 36.45 ppm. A significant difference is observed for C1 carbon shielding calculated with the B3LYP, BLYP, BP86, CAM-B3LYP, LC-BLYP, M06, PBE0, TPSSh and wB97x density functionals (156.38, 150.55, 155.09, 160.67, 165.91, 150.33, 87.57, 158.65 and 164.39 ppm). There is almost no influence of solvent polarity on proton shieldings (28.333 and 28.329 ppm for protons of C1-methyl in the gas phase and water). N9 shielding is significantly more sensitive to the environment, and the corresponding shieldings in the gas phase and chloroform are −1.01 vs. 4.32 ppm. As expected, a saturation effect is seen, and a further increase in polarity has a markedly smaller impact on N9 shielding (6.62 ppm in water).

Oxygen is the most sensitive nucleus to the polarity of the environment. The corresponding O2 shieldings calculated with B3LYP in the gas phase, chloroform and water are −6.21, 11.80 and 20.08 ppm, respectively. The sensitivity to environment polarity for other functionals is similar. In conclusion, from the results gathered in Table S1, we notice a significantly stronger sensitivity of oxygen-17 and nitrogen-15 nuclei to the solvent polarity than that for carbon-13 and hydrogen-1.

### 3.3. Nuclear Magnetic Shieldings of Reference Molecules

In order to discuss multinuclear chemical shifts in caffeine, the nuclear magnetic shieldings of the studied reference compounds (TMS, H_2_O and CH_3_NO_2_), calculated by nine density functionals in four environments, will be considered first. For brevity, these data are gathered in Table S2 in Supplementary Materials.

It is important to notice that the nuclear magnetic shieldings of the studied reference molecules depend both on the density functional and solvent used. Thus, the proton shieldings of TMS calculated with the B3LYP functional in the gas phase, chloroform, DMSO and water are 31.737, 31.733, 31.732 and 31.730 ppm. It changes somehow more for the used density functional, starting from B3LYP to wB97X (results in vacuum): 31.737, 31.498, 31.297, 31.747, 31.682, 31.978, 31.587, 31.768 and 31.707 ppm. The carbon shieldings of TMS, calculated by the M06 and LC-BLYP functionals in the gas phase, are 178.17 and 192.81 ppm, respectively. In the case of oxygen, the corresponding gas phase shieldings differ more (309.01 and 336.91 ppm). Before calculating the ^17^O NMR chemical shift, the theoretical reference value needs to include the gas-to-liquid shift of −36.1 ppm [62].

### 3.4. ^1^H, ^13^C, ^15^N and ^17^O Chemical Shifts in Caffeine

The chemical shifts in caffeine calculated using the theoretical nuclear magnetic shieldings of the reference molecules are included in Table S3 in Supplementary Materials. Since chemical shifts are relative values, we could expect some beneficial systematic error cancelation between the nuclear shieldings of caffeine and reference molecules. In addition, as for caffeine shielding constants (see Table S1), it is apparent from Table S3 that the theoretical chemical shifts are also susceptible to solvent polarity and the density functional used.

Nitrogen-15 is the most sensitive caffeine nucleus to the solvent effect. Its B3LYP results in vacuum and water are −151.84 ppm and −164.48 ppm. On the other hand, the corresponding M06 results are also sensitive to solvent polarity and are −165.11 and −178.10 ppm, respectively.

To assess the performance of the selected density functionals in predicting caffeine multinuclear chemical shifts, in Table S4 in Supplementary Materials, we included their deviations from experimental data [25], measured in CDCl_3_. These data allow for the inspection of accuracy for each atom, as well as some statistics by calculating RMS deviations for carbon, protons, nitrogen and oxygen. For oxygen, we used the same parameter (RMS) for consistency only (averaging two numbers produce similar values). Looking at the deviation of chemical shifts in Table S4, one could notice a kind of saturation effect upon changing solvent polarity.

The deviations of predicted ^13^C chemical shifts, calculated with nine density functionals in vacuum, chloroform and water from the experiment, are gathered in Table 1. It is evident that most functionals overestimate C4 and C6 chemical shifts in vacuum by up to 15 ppm. However, TPSSh performs significantly better in vacuum with deviations from 1 to 4 ppm. On the other hand, in water, the deviations are more significant (from 2 to 5 ppm).

Table 2 presents gathered deviations of predicted ^1^H chemical shifts, calculated with nine density functionals in vacuum and water from the experiment. As expected, these deviations are smaller and in most cases below 0.5 ppm (from 0.02 to 0.7 ppm in vacuum and 0.001 to 0.2 ppm in chloroform). 

It is worth noticing that theoretical ^15^N chemical shift deviations from the experiments are significantly larger (up to 30 ppm in vacuum and 50 ppm in chloroform; see Table 3). A similar trend is observed for oxygen-17. It is worth noting that O6 is predicted much more accurately than O2 (see Table 4). 

The RMS values for the DFT predicted multinuclear chemical shifts in caffeine, calculated from individual deviations (see Table 1, Table 2, Table 3 and Table 4), are gathered in Table 5. Bold font marks the best results (smallest RMS). These results indicate clear differences in the performance of the selected density functionals. Additionally, it is difficult to select one functional that performs the best for all nuclei in caffeine.

**Figure 3:**
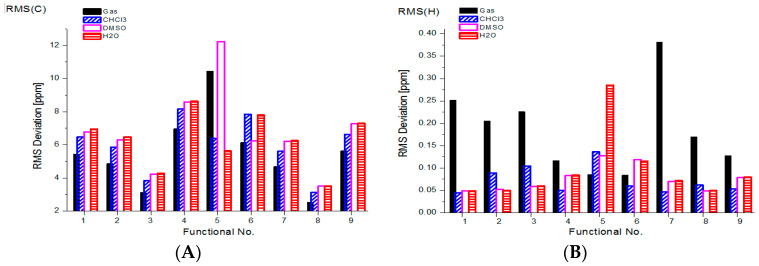

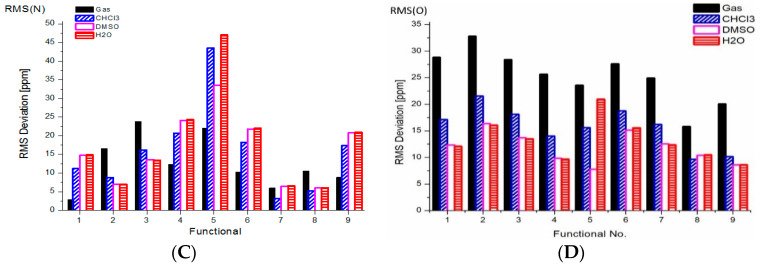
RMS deviations (in ppm) of chemical shifts in (**A**) ^13^C, (**B**) ^1^H, (**C**) ^15^N and (**D**) ^17^O calculated with selected density functionals for free caffeine in vacuum and in solution (numbers are assigned to used functionals in computational part).

A better presentation of the DFT performance of caffeine multinuclear chemical shift prediction is apparent from Figure 3A–D.

It is apparent from Figure 3A that the majority of density functionals predict smaller RMS values of the ^13^C shieldings of caffeine in vacuum and a low-polarity environment. Moreover, a kind of saturation of RMS is observed along with increased solvent polarity. Additionally, the worst and irregular (scattered) behavior with respect to solvent polarity performance are shown in functionals 5 and 6 (TPSSh and BP86). On the other hand, the best overall performance in vacuum and solution is shown in functionals 8 (TPSSh) and 3 (BP86) with RMS(C) below 3.5 and 4.3 ppm, respectively. The other density functionals yield markedly worse results (RMS(C) of 6–8 ppm).

The ^1^H parameters of caffeine were predicted with significantly smaller RMS values (Figure 3B). Thus, in the gas phase, the RMS(H) calculated with nine functionals was in the range from 0.09 to 0.38 ppm and in water from 0.05 to 0.12 ppm. Interestingly, the best results in solution were observed for B3LYP (RMS < 0.05 ppm).

The predicted N-15 chemical shifts are significantly less accurate (Figure 3C). In several cases, the RMS(N) increases along with solvent polarity. The worst results are predicted with the LC-BLYP density functional (RMS(N) is about 50 ppm), and the best nitrogen shieldings are calculated with PBE0 and TPSSh (RMS (N) from 3 to 7 ppm).

The corresponding caffeine’s results for oxygen-17 are presented in Figure 3D. The worst results are observed for in vacuum (RMS(O) from 16 to 33 ppm), and they decrease with solvent polarity. As before, LC-BLYP produces irregular deviations (RMS(O) from 8 to 24 ppm), and the best performance in all analyzed environments is shown by TPSSh (RMS(O) of about 10 ppm).

In addition, to assess the prediction efficiency of the caffeine chemical shift, the corresponding RMS deviations are recalculated below as the percentage of the typical range of 200 ppm for ^13^C, 10 ppm for ^1^H, 900 ppm for ^15^N and about 1200 ppm for ^17^O. Thus, the deviations (%) of a typical range of chemical shifts in caffeine are about 2, 0.9–4, 0.3–0.8 and 1% for the ^13^C, ^1^H, ^15^N and ^17^O NMR chemical shifts, respectively. These results indicate that when using the selected density functionals individually for a single nucleus combined with the aug-cc-pVTZ basis set, we can consider the predicted chemical shifts in caffeine as fairly accurate.

### 3.5. Indirect Spin–Spin Coupling Constants (SSCCs) of Caffeine in Vacuum and Solution

In our recent study on one-bond ^15^N-^1^H couplings, we observed a very good agreement between theoretical and experimental SSCC values for some sp^2^-hybridized nitrogen atoms of Schiff bases, enaminones and similar compounds using the B3LYP density functional and an efficient “mixed” basis set approach [60,63]. This approach allows for a faster calculation of SSCCs, in particular the Fermi component, which often decides the total value of J-coupling. In the subsequent part of this study, in analogy to nuclear shieldings, selected SSCC parameters were predicted with the above-used density functionals and solvents (see Table 6).

In Table S5, selected ^13^C-^1^H and ^15^N-^1^H coupling constants are gathered (19 different nuclei positions), calculated with nine density functionals in four environments. As expected, the one-bond ^13^C-^1^H couplings in three different methyl groups are very large in comparison to couplings through several bonds, but the largest is C8-H8 (the corresponding B3LYP magnitudes for these one-bond C-H couplings are about 145 and 216 Hz). The SSCC parameters are strongly dependent on the used density functional. Thus, the C8-H8 couplings, calculated in the gas phase by B3LYP, BLYP, BP86, CAM-B3LYP, LC-BLYP, M06, PBE0, TPSSh and wB97X, are 215.8, 222.0, 200.5, 211.0, 202.8, 239.8, 202.6, 228.3 and 190.6 Hz, respectively. However, the impact of the solvent is significantly smaller. For example, the one-bond C8-H8 couplings predicted in vacuum, chloroform, DMSO and water are 215.84, 219.09, 220.47 and 220.54 Hz, respectively.

There are 19 available experimental J-couplings, and Table S6 shows the deviations of the DFT calculated couplings from experimental values [25]. As expected, depending on the functional used, the largest deviations between theoretical and experimental couplings in the gas phase are for C8-H8 (from 1 to 40 Hz) and one-bond C-H in methyl groups (from 0.05 to 17 Hz; see Table S6). For an easier assessment of DFT performance in the prediction of one-bond C-H couplings, Table S6 compiles the deviations of theoretical C-H couplings from the experiment and the corresponding RMS values. Comparing the RMS values in vacuum, it is clear that CAM-B3LYP performs the best, B3LYP is the second best one and M06 yields the worst results (Table 6). The corresponding RMS values for these functionals are 0.66, 4.10 and 20.2 Hz.

**Figure 4:**
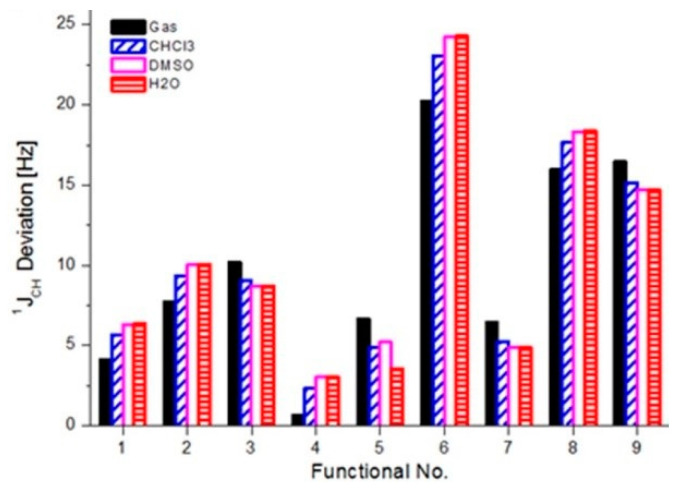
RMS deviations of DFT calculated caffeine’s ^1^J(C-H) in different environments from experiment [25] (numbers are assigned to used functionals in computational part).

The performance of an individual density functional in predicting one-bond C-H couplings in different environments is also clearly visible in Figure 4. The worst performance is shown by M06 (RMS reaching 25 Hz), and the most accurate is CAM-B3LYP (RMS from 0.66 to 3.05 Hz). Additionally, B3LYP, LC-BLYP and PBE0 also perform well (RMS of 4.10, 6.64 and 6.42 Hz in vacuum). Interestingly, TPSSh seems to be the best one in predicting caffeine chemical shifts, but it is inappropriate for ^1^J_C-H_ couplings (RMS close to 20 Hz). In addition, wB97X is also very bad (RMS of 16.44 Hz).

The deviations of ^2^J_NH_ couplings from the experiment [25] and the corresponding RMS values are collected in Table S6. In this case, the smallest RMS is obtained for TPSSh in water (1.06 Hz) and the worst one for M06 (1.56 Hz in vacuum). The best and worst methods for the prediction of ^3^J_CH_ are BP86 and M06, respectively (RMS of 0.31 and 0.72 in vacuum; see Table S6). The BP86 density functional is the best in reproducing experimental ^4^J_CH_ (deviation of 0.07 Hz in vacuum, Table S6).

The calculated SSCC parameters of caffeine point toward the necessity of using different density functionals for each type of coupling constant and chemical shift [64]. This result points toward the semi-empirical nature of DFT methodology.

## 4. Conclusions

A combined experimental and theoretical study on the NMR spectroscopy of caffeine is reported on. For the first time, we report on the experimental ^17^O NMR spectra of caffeine in solution. The assignment of two oxygen-17 peaks of caffeine was supported by systematic DFT calculations. The solvent effect was included via an implicit PCM model, and both multinuclear isotropic magnetic shieldings (chemical shifts) and indirect spin–spin coupling constants were calculated in vacuum, chloroform, DMSO and water. Benchmark GIAO calculations using nine functionals (B3LYP, BLYP, BP86, CAM-B3LYP, LC-BLYP, M06, PBE0, TPSSh, wB97x) and a large aug-cc-pVTZ basis set predicted fairly reasonable chemical shifts for the ^13^C, ^1^H, ^15^N and ^17^O nuclei. The following smallest RMS deviations for caffeine nuclei in chloroform were calculated: ^13^C with TPSSh and BP86 of 3.14 and 3.84 ppm; ^1^H with B3LYP and PBE0 of 0.044 and 0.047 ppm; ^15^N with PBE0 and TPSSh of 3.14 and 5.22 ppm; and ^17^O with LC-BLYP and TPSSh of 15.62 and 9.66 ppm.

The prediction of ^1^J_CH_ coupling was more efficient using CAM-B3LYP/aug-cc-pVTZ (mixed) calculations (RMS deviation of 0.66 and 2.31 Hz in vacuum and chloroform).

It is evident from the obtained data that no single density functional works best for all tested NMR parameters of purines. Therefore, an optimal methodology for calculating the chemical shifts and J-couplings of caffeine and similar molecules has been developed. We suggest using the TPSSh and BP86 density functionals to obtain reliable ^13^C NMR data, B3LYP and PBE0 for ^1^H, PBE0 and TPSSh for ^15^N, and LC-BLYP and TPSSh for ^17^O, suitable for studies of purines and similar natural products.

## Figures and Tables

**Table 1:** Deviations of theoretical ^13^C chemical shifts (in ppm) of caffeine, calculated with selected density functionals in vacuum and chloroform, from experiment.

Atom	B3LYP	BLYP	BP86	CAM-B3LYP	LC-BLYP	M06	PBE0	TPSSh	wB97X	Exp. ^a^
**Vacuum**										
C1	1.080	1.692	0.811	0.346	−0.605	0.334	0.581	1.804	0.295	**27.5**
C2	6.253	4.532	1.563	9.001	13.998	6.524	5.334	1.716	7.398	**151.3**
C3	1.627	2.548	1.696	0.807	−0.346	3.116	1.445	2.351	0.683	**29.3**
C4	8.287	6.597	3.678	10.365	14.488	10.207	7.109	2.623	8.213	**148.3**
C5	7.573	8.659	6.354	7.168	8.305	6.351	6.285	3.425	5.000	**107.1**
C6	6.281	4.020	1.092	9.857	15.942	5.605	5.693	1.063	8.666	**154.9**
C7	3.510	4.627	3.802	2.606	1.201	3.683	3.045	4.100	2.276	**33.2**
C8	3.713	1.160	−0.899	6.479	11.815	7.554	3.344	−1.436	4.948	**141.2**
**Chloroform**										
C1	1.530	2.270	1.322	0.913	−10.130	2.070	0.921	2.206	0.608	**27.5**
C2	7.818	6.102	2.987	10.780	2.853	8.672	6.776	3.083	8.939	**151.3**
C3	2.249	3.340	2.394	1.511	−10.136	3.647	1.952	2.904	1.127	**29.3**
C4	8.903	7.305	4.320	11.117	−0.817	11.548	7.618	3.166	8.710	**148.3**
C5	8.276	9.601	7.222	7.898	−5.067	6.046	6.792	4.046	5.394	**107.1**
C6	7.570	5.320	2.247	11.370	3.379	8.788	6.842	2.125	9.883	**154.9**
C7	4.213	5.523	4.607	3.384	−8.568	4.819	3.631	4.841	2.837	**33.2**
C8	6.613	4.141	2.042	9.510	0.988	11.309	6.146	1.336	7.701	**141.2**

^a^ From ref. [25].

**Table 2:** Deviations of theoretical ^1^H chemical shifts (in ppm) of caffeine, calculated with selected density functionals in vacuum and chloroform, from experiment.

Atom	B3LYP	BLYP	BP86	CAM-B3LYP	LC-BLYP	M06	PBE0	TPSSh	wB97X	Exp. ^a^
**Vacuum**										
H1	0.034	0.032	0.016	0.060	0.074	−0.027	−0.712	0.032	0.008	**3.37**
H3	0.012	0.018	0.001	0.026	0.026	0.034	−0.018	0.002	−0.018	**3.55**
H7	−0.114	−0.128	−0.146	−0.100	−0.098	−0.103	−0.138	−0.144	−0.142	**4.01**
H8	−0.272	−0.388	−0.428	−0.200	−0.116	−0.125	−0.229	−0.305	−0.213	**7.58**
**Chloroform**										
H1	0.037	0.046	0.027	0.058	−0.093	−0.021	−0.001	0.030	0.004	**3.37**
H3	0.030	0.048	0.028	0.039	−0.143	−0.008	−0.001	0.017	−0.009	**3.55**
H7	−0.068	−0.075	−0.096	−0.055	−0.209	−0.058	−0.092	−0.101	−0.102	**4.01**
H8	−0.031	−0.146	−0.181	0.044	−0.033	0.103	0.016	−0.063	0.030	**7.58**

^a^ From ref. [25].

**Table 3:** Deviations of theoretical ^15^N chemical shifts deviations (in ppm) of caffeine, calculated with selected density functionals in vacuum and chloroform, from experiment.

Atom	B3LYP	BLYP	BP86	CAM-B3LYP	LC-BLYP	M06	PBE0	TPSSh	wB97X	Exp. ^a^
**Vacuum**										
N1	−1.509	20.745	27.929	−12.802	−24.166	−11.534	6.479	12.379	−9.465	**−231.3**
N3	−3.515	18.243	26.339	−15.655	−27.967	−13.999	4.612	12.752	−11.581	**−268.4**
N7	−4.194	11.793	19.855	−12.064	−19.942	−9.574	4.264	8.604	−7.714	**−227.2**
N9	0.372	14.039	20.120	−7.034	−13.512	−0.371	7.832	7.450	−5.791	**−151.2**
**Chloroform**										
N1	−9.994	11.984	19.668	−21.070	−44.037	−20.328	−1.438	4.479	−17.412	**−231.3**
N3	−10.168	11.338	19.897	−22.139	−44.485	−20.723	−1.531	6.626	−17.865	**−268.4**
N7	−9.950	6.074	14.688	−17.768	−39.357	−15.345	−0.915	3.471	−13.224	**−227.2**
N9	−14.033	−0.196	6.729	−21.667	−45.876	−15.719	−5.836	−5.758	−20.195	**−151.2**

^a^ From ref. [25].

**Table 4:** Deviations of theoretical ^17^O chemical shifts (in ppm) of caffeine, calculated with selected density functionals in vacuum and chloroform, from experiment.

Atom	B3LYP	BLYP	BP86	CAM-B3LYP	LC-BLYP	M06	PBE0	TPSSh	wB97X	Exp. ^a^
**Vacuum**										
O2	37.302	41.158	37.013	33.858	31.265	38.317	35.085	22.308	27.429	**258.602**
O6	16.501	21.502	15.523	13.015	11.691	8.316	16.318	1.443	7.288	**306.269**
**Chloroform**										
O2	23.767	28.623	25.079	19.778	−4.859	23.993	22.112	10.400	13.724	**258.602**
O6	4.838	10.454	5.225	1.438	−21.545	6.837	5.040	−8.855	−4.287	**306.269**

^a^ This work.

**Table 5:** Summarized RMS deviations of caffeine ^15^N, ^13^C, ^17^O and ^1^H chemical shifts ^a^ calculated with selected density functionals in vacuum and solution.

Label	Solvent	Vacuum	Chloroform	DMSO	Water
	**Functional**	**^13^C**			
1	B3LYP	5.414	6.472	6.783	6.951
2	BLYP	4.839	5.863	6.298	6.485
3	BP86	3.089	3.835	4.219	4.273
4	CAM-B3LYP	6.937	8.153	8.591	8.633
5	LC-BLYP	10.431	6.380	12.241	5.643
6	M06	6.113	7.835	6.229	7.798
7	PBE0	4.661	5.618	6.224	6.260
**8**	**TPSSh**	**2.508**	**3.141**	**3.504**	**3.514**
9	wB97X	5.618	6.629	7.282	7.314
		**^1^H**			
1	B3LYP	0.149	0.044	0.049	0.049
2	BLYP	0.205	0.089	0.052	0.050
3	BP86	0.226	0.104	0.059	0.060
4	CAM-B3LYP	0.117	0.050	0.083	0.084
5	LC-BLYP	0.085	0.136	0.128	0.285
6	M06	0.084	0.060	0.119	0.115
7	PBE0	0.381	0.047	0.070	0.072
**8**	**TPSSh**	**0.170**	**0.062**	**0.049**	**0.050**
9	wB97X	0.128	0.053	0.079	0.080
		**^15^N**			
1	B3LYP	2.844	11.171	14.716	14.896
2	BLYP	16.578	8.791	7.038	6.989
3	BP86	23.837	16.153	13.603	13.477
4	CAM-B3LYP	12.288	20.732	24.126	24.296
5	LC-BLYP	22.059	43.508	33.519	47.007
6	M06	10.257	18.202	21.817	21.997
7	PBE0	5.974	**3.135**	6.434	6.604
**8**	**TPSSh**	**10.552**	**5.224**	**5.958**	**6.043**
9	wB97X	8.898	17.357	20.737	20.910
		**^17^O**			
1	B3LYP	28.842	17.150	12.361	12.129
2	BLYP	32.835	21.547	16.381	16.105
3	BP86	28.381	18.114	13.725	13.503
4	CAM-B3LYP	25.649	14.022	9.844	9.675
5	LC-BLYP	23.603	15.617	**7.785**	20.971
6	M06	27.616	18.756	15.124	15.564
7	PBE0	24.921	16.228	12.572	12.385
**8**	**TPSSh**	**15.807**	**9.658**	**10.394**	**10.512**
9	wB97X	20.068	10.166	8.627	**8.646**

^a^ The best results are marked in bold.

**Table 6:** RMS deviations of ^1^J_C-H_ spin–spin coupling constants, calculated with selected functionals in gas phase and solutions, from experiment [25].

Functional	Vacuum	Chloroform	DMSO	Water
B3LYP	4.096	5.667	6.323	6.357
BLYP	7.727	9.364	10.043	10.078
BP86	10.178	9.084	8.724	8.708
**CAM-B3LYP**	**0.658**	**2.305**	**3.012**	**3.048**
LC-BLYP	6.637	4.904	5.206	3.571
M06	20.233	23.050	24.232	24.290
PBE0	6.421	5.239	4.882	4.867
TPSSh	15.948	17.640	18.334	18.369
wB97X	16.443	15.164	14.696	14.675

## Data Availability

All data are available upon request from the authors.

## Supplementary Materials

The following supporting information can be downloaded at https://www.mdpi.com/article/10.3390/molecules29184382/s1, Table S1: Theoretical nuclear magnetic shieldings (in ppm) of caffeine calculated with selected functionals combined with aug-cc-pVTZ basis set; Table S2: Theoretical nuclear magnetic shieldings (in ppm) of reference compounds calculated with selected functionals combined with aug-cc-pVTZ basis set; Table S3: Theoretical chemical shifts (in ppm) of caffeine calculated with selected functionals combined with aug-cc-pVTZ basis set; Table S4: Deviations of caffeine theoretical chemical shifts from experimenta (in ppm) combined with aug-cc-pVTZ basis set; Table S5: Indirect spin-spin coupling constants (in Hz) of caffeine calculated with selected functionals combined with “mixed” aug-cc-pVTZ basis set; Table S6: Deviations of theoretical spin-spin coupling constants from available experimental values (in Hz) of caffeine calculated with selected density functionals combined with “mixed” aug-cc-pVTZ basis set; Table S7: Deviations of selected 1JC-H theoretical spin-spin coupling constants from available experimental valuesa (in Hz) of caffeine calculated with selected density functionals combined with “mixed” aug-cc-pVTZ basis set; Appendix I: Cartesian cordinates of caffeine calculated at B3LYP/aug-cc-pVTZ level of theory in vacuum and solvents in PCM model; Appendix II: Cartesian cordinates of caffeine calculated at BLYP/aug-cc-pVTZ level of theory in vacuum and solvents in PCM model; Appendix III: Cartesian cordinates of caffeine calculated at BP86/aug-cc-pVTZ level of theory in vacuum and solvents in PCM model; Appendix IV: Cartesian cordinates of caffeine calculated at CAM-B3LYP/aug-cc-pVTZ level of theory in vacuum and solvents in PCM model; Appendix V: Cartesian cordinates of caffeine calculated at LC-BLYP/aug-cc-pVTZ level of theory in vacuum and solvents in PCM model; Appendix VI: Cartesian cordinates of caffeine calculated at M06/aug-cc-pVTZ level of theory in vacuum and solvents in PCM model; Appendix VII: Cartesian cordinates of caffeine calculated at PBE0/aug-cc-pVTZ level of theory in vacuum and solvents in PCM model; Appendix VIII: Cartesian cordinates of caffeine calculated at TPSSh/aug-cc-pVTZ level of theory in vacuum and solvents in PCM model; Appendix IX: Cartesian cordinates of caffeine calculated at wB97X/aug-cc-pVTZ level of theory in vacuum and solvents in PCM model.
