# Supplementary material for: Caffeine—Legal Natural Stimulant with Open Research Perspective: Spectroscopic and Theoretical Characterization
Source: Molecules. 2024 Sep 14;29(18):4382. doi: 10.3390/molecules29184382 (PMC11434362; doi:10.3390/molecules29184382)
Supplement: Supplementary file 1 [file molecules-29-04382-s001.zip › molecules-3132911-supplementary.pdf]

SUPPORTING INFORMATION FOR

Caffeine – legal natural stimulant with open research perspective: spectroscopic and theoretical characterization

Teobald Kupka*, Natalina Makieieva*, Michał Jewgiński, Magdalena Witek,
Barbara Blicharska, Oimahmad Rahmonov, Karel Doležal and Tomáš Pospíšil

Table S1. Theoretical nuclear magnetic shieldings (in ppm) of caffeine calculated with selected functionals combined with aug-cc-pVTZ basis set[a]

Nuclei	**B3LYP**				**BLYP**			
	**Gas**	**$CHCl_3$**	**DMSO**	**H_2O**	**Gas**	**$CHCl_3$**	**DMSO**	**H_2O**
**C1**	156.377	156.293	156.262	156.261	150.546	150.457	150.429	150.428
**C2**	27.404	26.206	25.722	25.697	23.906	22.825	22.382	22.359
**C3**	154.030	153.774	153.681	153.677	147.890	147.587	147.480	147.475
**C4**	28.370	28.120	28.029	28.025	24.841	24.622	24.550	24.546
**C5**	70.284	69.947	69.761	69.751	63.979	63.526	63.296	63.283
**C6**	23.776	22.853	22.459	22.438	20.818	20.007	19.659	19.640
**C7**	148.247	147.910	147.787	147.781	141.911	141.504	141.354	141.347
**C8**	40.044	37.510	36.503	36.450	37.378	34.886	33.886	33.835
**H1av**	28.333	28.327	28.329	28.329	28.096	28.091	28.093	28.093
**H3av**	28.174	28.153	28.149	28.149	27.929	27.909	27.905	27.905
**H7av**	27.840	27.792	27.775	27.774	27.616	27.571	27.556	27.555
**H8**	24.429	24.184	24.085	24.080	24.306	24.073	23.977	23.972
**N1**	80.969	80.378	80.240	80.234	65.002	64.597	64.570	64.571
**N3**	120.075	117.652	116.675	116.624	104.603	102.343	101.451	101.405
**N7**	79.554	76.234	75.033	74.974	69.854	66.406	65.135	65.072
**N9**	-1.012	4.316	6.509	6.622	-8.393	-3.323	-1.219	-1.109
**O2**	-6.210	11.795	19.655	20.077	-16.597	0.936	8.678	9.097
**O6**	-33.076	-16.943	-9.718	-9.329	-44.607	-28.562	-21.355	-20.967
Nuclei	**BP86**				**CAM-B3LYP**			
**C1**	155.085	155.034	155.014	155.013	160.672	160.577	160.539	160.537
**C2**	30.533	29.569	29.176	29.155	28.218	26.911	26.386	26.359
**C3**	152.400	152.162	152.076	152.072	158.412	158.180	158.096	158.092
**C4**	31.419	31.236	31.179	31.176	29.853	29.573	29.470	29.464
**C5**	69.942	69.534	69.327	69.316	74.250	73.992	73.841	73.832
**C6**	27.404	26.709	26.410	26.394	23.761	22.721	22.275	22.251
**C7**	146.395	146.050	145.919	145.912	152.713	152.406	152.293	152.288
**C8**	43.095	40.615	39.615	39.564	40.839	38.281	37.272	37.220
**H1av**	27.910	27.903	27.904	27.904	28.318	28.312	28.314	28.314
**H3av**	27.746	27.722	27.717	27.717	28.171	28.151	28.148	28.148
**H7av**	27.432	27.387	27.370	27.369	27.837	27.785	27.768	27.767
**H8**	24.144	23.901	23.801	23.795	24.368	24.116	24.014	24.009
**N1**	72.887	72.524	72.500	72.501	91.429	90.708	90.495	90.485
**N3**	111.578	109.395	108.542	108.498	131.381	128.877	127.856	127.803
**N7**	76.861	73.405	72.125	72.062	86.590	83.306	82.124	82.066
**N9**	0.597	5.364	7.335	7.437	5.560	11.205	13.522	13.643
**O2**	-9.735	6.969	14.302	14.698	1.696	19.953	27.902	28.329
**O6**	-35.912	-20.844	-14.100	-13.736	-25.127	-9.374	-2.304	-1.923

[a] methyl protons shieldings are averaged.

Table S1. Cont.

Nuclei	LC-BLYP				M06			
	Gas	$CHCl_3$	DMSO	H_2O	Gas	$CHCl_3$	DMSO	H_2O
**C1**	165.910	173.482	165.755	173.438	150.333	149.641	150.307	150.307
**C2**	27.507	36.699	25.469	36.151	20.343	19.239	18.482	18.454
**C3**	163.851	171.688	163.574	171.612	145.751	146.264	145.347	145.341
**C4**	30.018	43.369	29.571	43.257	19.660	19.362	19.394	19.390
**C5**	77.400	88.820	77.081	88.712	64.716	66.065	64.244	64.234
**C6**	21.963	32.574	20.260	32.125	17.663	15.522	16.556	16.536
**C7**	158.404	166.221	158.035	166.139	141.284	141.192	140.950	140.946
**C8**	39.790	48.665	36.129	47.615	29.413	26.701	25.816	25.763
**H1av**	28.238	28.044	28.235	28.047	28.635	28.598	28.632	28.632
**H3av**	28.107	27.913	28.085	27.910	28.394	28.405	28.365	28.365
**H7av**	27.770	27.5196	27.698	27.500	28.071	27.996	28.002	28.001
**H8**	24.218	23.773	23.851	23.659	24.522	24.265	24.160	24.154
**N1**	103.480	114.484	102.356	114.231	77.720	77.184	77.227	77.226
**N3**	144.380	152.032	140.767	150.991	117.285	114.679	114.277	114.233
**N7**	95.155	105.704	90.654	104.528	71.660	68.100	67.294	67.237
**N9**	12.725	36.223	21.191	38.671	-13.543	-7.525	-5.667	-5.546
**O2**	10.939	50.711	37.617	58.978	-24.008	-5.318	1.235	1.648
**O6**	-17.154	19.730	5.014	26.718	-41.673	-35.828	-18.254	-17.892
Nuclei	**PBE0**				**TPSSh**			
**C1**	161.480	161.408	161.380	161.378	158.740	158.672	158.646	158.645
**C2**	32.927	31.754	31.282	31.257	35.028	33.996	33.581	33.559
**C3**	158.816	158.577	158.491	158.487	156.393	156.174	156.100	156.097
**C4**	34.153	33.912	33.825	33.821	37.121	36.912	36.844	36.840
**C5**	76.176	75.937	75.800	75.792	77.520	77.232	77.072	77.063
**C6**	28.968	28.087	27.715	27.695	32.081	31.353	31.046	31.029
**C7**	153.316	152.999	152.879	152.873	150.745	150.338	150.183	150.176
**C8**	45.018	42.483	41.4673	41.415	48.280	45.843	44.867	44.816
**H1av**	28.929	28.208	28.208	28.208	28.366	28.359	28.361	28.361
**H3av**	28.055	28.028	28.022	28.021	28.216	28.192	28.186	28.186
**H7av**	27.714	27.659	27.640	27.639	27.902	27.850	27.831	27.830
**H8**	24.235	23.981	23.877	23.872	24.493	24.242	24.140	24.135
**N1**	87.570	86.973	86.830	86.824	87.383	86.868	86.760	86.756
**N3**	126.537	124.166	123.219	123.170	124.110	121.822	120.909	120.861
**N7**	85.685	82.350	81.150	81.091	87.058	83.777	82.585	82.526
**N9**	6.117	11.272	13.370	13.479	12.212	17.006	18.976	19.078
**O2**	-1.533	15.718	23.185	23.585	8.401	24.835	32.007	32.392
**O6**	-30.433	-14.877	-7.962	-7.591	-18.401	-3.578	3.039	3.394
Nuclei	**wB97X**							
**C1**	164.387	164.297	164.255	164.253				
**C2**	33.483	32.166	31.634	31.607				
**C3**	162.199	161.979	161.895	161.891				
**C4**	35.668	35.396	35.300	35.295				
**C5**	80.082	79.911	79.796	79.789				
**C6**	28.615	27.622	27.176	27.150				
**C7**	156.705	156.369	156.251	156.246				
**C8**	46.034	43.505	42.511	42.460				
**H1av**	28.329	28.321	28.322	28.322				
**H3av**	28.175	28.154	28.150	28.150				
**H7av**	27.839	27.787	27.769	27.769				
**H8**	24.340	24.086	23.983	23.978				
**N1**	99.621	98.839	98.593	98.581				
**N3**	138.837	136.391	135.392	135.340				
**N7**	93.770	90.551	89.389	89.332				
**N9**	15.847	21.521	23.851	23.972				
**O2**	9.822	27.496	35.193	35.606				
**O6**	-17.704	-2.161	4.627	4.981				

Table S2. Theoretical nuclear magnetic shieldings (in ppm) of reference compounds calculated with selected functionals combined with aug-cc-pVTZ basis set

Compound	*Nuclei*	**B3LYP**				**BLYP**			
		**Gas**	**$CHCl_3$**	**DMSO**	**H_2O**	**Gas**	**$CHCl_3$**	**DMSO**	**H_2O**
TMS	**C**	184.957	185.323	185.308	185.472	179.738	180.227	180.393	180.575
	**H**	31.737	31.733	31.732	31.730	31.498	31.507	31.509	31.506
CH_3NO_2	**N**	-151.840	-160.917	-164.309	-164.480	-145.554	-154.719	-158.186	-158.362
H_2O	**O**	325.794	330.264	331.817	331.893	319.263	324.260	326.002	326.088
Compound	*Nuclei*	**BP86**				**CAM-B3LYP**			
TMS	**C**	183.396	183.856	184.031	184.076	188.518	188.991	189.090	189.117
	**H**	31.297	31.300	31.305	31.296	31.747	31.740	31.740	31.738
CH_3NO_2	**N**	-130.484	-139.108	-142.363	-142.535	-152.674	-161.662	-165.007	-165.172
H_2O	**O**	321.980	326.750	328.414	328.495	330.256	334.434	335.883	335.954
Compound	*Nuclei*	**LC-BLYP**				**M06**			
TMS	**C**	192.805	190.852	193.400	193.409	178.167	179.210	176.940	178.722
	**H**	31.682	31.320	31.672	31.673	31.978	31.948	31.964	31.946
CH_3NO_2	**N**	-151.987	-160.853	-164.126	-164.289	-165.114	-174.445	-177.927	-178.102
H_2O	**O**	336.906	340.554	341.817	341.879	309.012	313.377	314.884	314.957
Compound	*Nuclei*	**PBE0**				**TPSSh**			
TMS	**C**	189.561	189.829	190.140	190.161	188.044	188.378	188.526	188.522
	**H**	31.587	31.577	31.573	31.573	31.768	31.759	31.763	31.762
CH_3NO_2	**N**	-137.251	-145.765	-148.933	-149.093	-131.538	-139.953	-143.108	-143.267
H_2O	**O**	328.254	332.532	334.020	334.093	325.411	329.937	331.517	331.595
Compound	*Nuclei*	**wB97X**							
TMS	**C**	192.181	192.406	192.749	192.765				
	**H**	31.707	31.695	31.696	31.691				
CH_3NO_2	**N**	-141.144	-149.873	-153.123	-153.287				
H_2O	**O**	331.953	335.921	337.300	337.367				

Table S3. Theoretical chemical shifts (in ppm) of caffeine calculated with selected functionals combined with aug-cc-pVTZ basis set[a]

Nuclei	B3LYP				BLYP			
	**Gas**	**$CHCl_3$**	**DMSO**	**H_2O**	**Gas**	**$CHCl_3$**	**DMSO**	**H_2O**
**C1**	28.580	29.030	29.046	29.211	29.192	29.770	29.964	30.148
**C2**	157.553	159.118	159.585	159.775	155.832	157.402	158.011	158.217
**C3**	30.927	31.549	31.627	31.795	31.848	32.640	32.913	33.100
**C4**	156.587	157.203	157.278	157.447	154.897	155.605	155.843	156.029
**C5**	114.673	115.376	115.547	115.721	115.759	116.701	117.097	117.293
**C6**	161.181	162.470	162.849	163.034	158.920	160.220	160.734	160.935
**C7**	36.710	37.413	37.521	37.691	37.827	38.723	39.039	39.229
**C8**	144.913	147.813	148.805	149.022	142.360	145.341	146.507	146.741
**H1av**	3.404	3.407	3.403	3.401	3.402	3.416	3.416	3.413
**H3av**	3.562	3.580	3.583	3.581	3.568	3.598	3.605	3.601
**H7av**	3.896	3.942	3.957	3.955	3.882	3.935	3.953	3.951
**H8**	7.308	7.549	7.647	7.650	7.192	7.434	7.532	7.534
**N1**	-232.809	-241.294	-244.549	-244.715	-210.556	-219.316	-222.756	-222.933
**N3**	-271.915	-278.568	-280.984	-281.104	-250.157	-257.062	-259.637	-259.767
**N7**	-231.394	-237.150	-239.342	-239.454	-215.407	-221.126	-223.320	-223.434
**N9**	-150.828	-165.233	-170.818	-171.102	-137.161	-151.396	-156.967	-157.253
**O2**	332.004	318.469	312.163	311.816	335.860	323.325	317.324	316.991
**O6**	358.870	347.207	341.535	341.223	363.871	352.823	347.357	347.054
Nuclei	**BP86**				**CAM-B3LYP**			
**C1**	28.311	28.822	29.016	29.063	27.846	28.413	28.551	28.580
**C2**	152.863	154.287	154.855	154.921	160.301	162.080	162.704	162.759
**C3**	30.996	31.694	31.955	32.004	30.107	30.811	30.994	31.025
**C4**	151.978	152.620	152.852	152.900	158.665	159.417	159.620	159.653
**C5**	113.454	114.322	114.703	114.761	114.268	114.998	115.249	115.286
**C6**	155.992	157.147	157.621	157.683	164.757	166.270	166.815	166.866
**C7**	37.002	37.807	38.112	38.164	35.806	36.584	36.797	36.830
**C8**	140.301	143.242	144.415	144.513	147.679	150.710	151.817	151.897
**H1av**	3.386	3.397	3.401	3.392	3.430	3.428	3.426	3.424
**H3av**	3.551	3.578	3.588	3.579	3.576	3.589	3.592	3.590
**H7av**	3.864	3.914	3.935	3.927	3.910	3.955	3.972	3.971
**H8**	7.153	7.399	7.504	7.501	7.380	7.624	7.725	7.729
**N1**	-203.371	-211.632	-214.863	-215.035	-244.102	-252.370	-255.501	-255.657
**N3**	-242.061	-248.503	-250.904	-251.033	-284.055	-290.539	-292.863	-292.974
**N7**	-207.345	-212.512	-214.488	-214.596	-239.264	-244.968	-247.131	-247.238
**N9**	-131.080	-144.471	-149.697	-149.972	-158.234	-172.867	-178.529	-178.814
**O2**	331.715	319.781	314.111	313.797	328.560	314.480	307.981	307.626
**O6**	357.892	347.594	342.513	342.232	355.384	343.807	338.187	337.877

[a] methyl protons shieldings are averaged.

Table S3. Cont.

Nuclei	LC-BLYP				M06			
	**Gas**	**$CHCl_3$**	**DMSO**	**H_2O**	**Gas**	**$CHCl_3$**	**DMSO**	**H_2O**
**C1**	26.895	17.370	27.645	19.970	27.834	29.570	26.633	28.414
**C2**	165.298	154.153	167.931	157.258	157.824	159.972	158.458	160.268
**C3**	28.954	19.164	29.826	21.797	32.416	32.947	31.593	33.381
**C4**	162.788	147.483	163.830	150.152	158.507	159.848	157.546	159.331
**C5**	115.405	102.033	116.319	104.697	113.451	113.146	112.696	114.487
**C6**	170.842	158.279	173.140	161.284	160.505	163.688	160.384	162.186
**C7**	34.401	24.632	35.365	27.270	36.883	38.019	35.990	37.775
**C8**	153.015	142.188	157.271	145.794	148.754	152.509	151.124	152.958
**H1av**	3.444	3.277	3.438	3.626	3.343	3.349	3.332	3.314
**H3av**	3.576	3.408	3.587	3.763	3.584	3.542	3.599	3.581
**H7av**	3.912	3.801	3.974	4.172	3.907	3.952	3.962	3.945
**H8**	7.465	7.547	7.822	8.013	7.455	7.683	7.804	7.792
**N1**	-255.466	-275.337	-266.482	-278.521	-242.834	-251.628	-255.155	-255.328
**N3**	-296.367	-312.885	-304.893	-315.280	-282.399	-289.123	-292.204	-292.335
**N7**	-247.142	-266.557	-254.780	-268.818	-236.774	-242.545	-245.221	-245.340
**N9**	-164.712	-197.076	-185.317	-202.960	-151.571	-166.919	-172.260	-172.556
**O2**	325.967	289.843	304.201	282.901	333.019	318.695	313.649	313.309
**O6**	354.060	320.824	336.803	315.161	350.685	349.206	333.138	332.849
Nuclei	**PBE0**				**TPSSh**			
**C1**	28.081	28.421	28.761	28.783	29.304	29.706	29.879	29.877
**C2**	156.634	158.076	158.859	158.904	153.016	154.383	154.945	154.963
**C3**	30.745	31.252	31.649	31.674	31.651	32.204	32.425	32.425
**C4**	155.409	155.918	156.315	156.341	150.923	151.466	151.682	151.682
**C5**	113.385	113.892	114.340	114.369	110.525	111.146	111.453	111.459
**C6**	160.593	161.742	162.425	162.466	155.963	157.025	157.480	157.493
**C7**	36.245	36.831	37.261	37.288	37.300	38.041	38.342	38.347
**C8**	144.544	147.346	148.673	148.747	139.764	142.536	143.659	143.706
**H1av**	2.658	3.369	3.366	3.365	3.402	3.400	3.403	3.402
**H3av**	3.532	3.549	3.552	3.552	3.552	3.567	3.577	3.576
**H7av**	3.872	3.918	3.934	3.935	3.866	3.909	3.932	3.932
**H8**	7.351	7.596	7.696	7.702	7.275	7.517	7.623	7.628
**N1**	-224.821	-232.738	-235.763	-235.917	-218.922	-226.821	-229.868	-230.023
**N3**	-263.788	-269.931	-272.152	-272.263	-255.648	-261.774	-264.017	-264.129
**N7**	-222.936	-228.115	-230.083	-230.184	-218.596	-223.729	-225.693	-225.793
**N9**	-143.368	-157.036	-162.303	-162.572	-143.750	-156.958	-162.084	-162.346
**O2**	329.787	316.814	310.835	310.508	317.010	305.102	299.510	299.203
**O6**	358.687	347.409	341.982	341.684	343.812	333.514	328.479	328.200
Nuclei	**wB97X**							
**C1**	27.795	28.108	28.494	28.512				
**C2**	158.698	160.239	161.115	161.158				
**C3**	29.983	30.427	30.854	30.874				
**C4**	156.513	157.010	157.449	157.470				
**C5**	112.100	112.494	112.953	112.976				
**C6**	163.566	164.783	165.573	165.614				
**C7**	35.476	36.037	36.498	36.519				
**C8**	146.148	148.901	150.239	150.305				
**H1av**	3.378	3.374	3.374	3.369				
**H3av**	3.532	3.542	3.546	3.542				
**H7av**	3.868	3.908	3.926	3.923				
**H8**	7.367	7.610	7.713	7.713				
**N1**	-240.765	-248.712	-251.715	-251.868				
**N3**	-279.981	-286.265	-288.515	-288.627				
**N7**	-234.914	-240.424	-242.512	-242.619				
**N9**	-156.991	-171.395	-176.973	-177.259				
**O2**	322.131	308.426	302.107	301.761				
**O6**	349.657	338.082	332.673	332.385				

Table S4. Deviations of caffeine theoretical chemical shifts from experiment[a] (in ppm) combined with aug-cc-pVTZ basis set[b,c]

Nuclei	B3LYP				BLYP			
	**Gas**	**$CHCl_3$**	**DMSO**	**H_2O**	**Gas**	**$CHCl_3$**	**DMSO**	**H_2O**
**C1**	-1.509	-9.994	-13.249	-13.415	1.692	2.270	2.464	2.648
**C2**	-3.515	-10.168	-12.584	-12.704	4.532	6.102	6.711	6.917
**C3**	-4.194	-9.950	-12.142	-12.254	2.548	3.340	3.613	3.800
**C4**	0.372	-14.033	-19.618	-19.902	6.597	7.305	7.543	7.729
**C5**	1.080	1.530	1.546	1.711	8.659	9.601	9.997	10.193
**C6**	6.253	7.818	8.285	8.475	4.020	5.320	5.834	6.035
**C7**	1.627	2.249	2.327	2.495	4.627	5.523	5.839	6.029
**C8**	8.287	8.903	8.978	9.147	1.160	4.141	5.307	5.541
RMS	**5.414**	**6.472**	**6.783**	**6.951**	**4.839**	**5.864**	**6.298**	**6.485**
**H1av**	0.034	0.037	0.033	0.031	0.032	0.046	0.046	0.043
**H3av**	0.012	0.030	0.033	0.031	0.018	0.048	0.0545	0.051
**H7av**	-0.114	-0.068	-0.053	-0.055	-0.128	-0.075	-0.057	-0.059
**H8**	-0.272	-0.031	0.067	0.070	-0.388	-0.146	-0.048	-0.047
RMS	**0.149**	**0.044**	**0.049**	**0.049**	**0.205**	**0.089**	**0.052**	**0.050**
**N1**	-1.509	-9.994	-13.249	-13.415	20.745	11.984	8.545	8.367
**N3**	-3.515	-10.168	-12.584	-12.704	18.243	11.338	8.763	8.633
**N7**	-4.194	-9.950	-12.142	-12.254	11.793	6.074	3.880	3.766
**N9**	0.372	-14.033	-19.618	-19.902	14.039	-0.196	-5.767	-6.053
RMS	**2.844**	**11.171**	**14.716**	**14.896**	**16.578**	**8.791**	**7.038**	**6.989**
**O2**	37.302	23.767	17.461	17.114	41.158	28.623	22.622	22.289
**O6**	16.501	4.838	-0.834	-1.147	21.502	10.454	4.988	4.685
RMS	**28.842**	**17.150**	**12.361**	**12.129**	**32.835**	**21.547**	**16.381**	**16.105**
Nuclei	**BP86**				**CAM-B3LYP**			
**C1**	0.811	1.322	1.516	1.563	0.346	0.913	1.051	1.080
**C2**	1.563	2.987	3.555	3.621	9.001	10.780	11.404	11.459
**C3**	1.696	2.394	2.655	2.704	0.807	1.511	1.694	1.725
**C4**	3.678	4.320	4.552	4.600	10.365	11.117	11.320	11.353
**C5**	6.354	7.222	7.603	7.661	7.168	7.898	8.149	8.186
**C6**	1.092	2.247	2.721	2.783	9.857	11.370	11.915	11.966
**C7**	3.802	4.607	4.912	4.964	2.606	3.384	3.597	3.630
**C8**	-0.899	2.042	3.215	3.313	6.479	9.510	10.617	10.697
RMS	**3.089**	**3.835**	**4.219**	**4.273**	**6.937**	**8.153**	**8.591**	**8.633**
**H1av**	0.016	0.027	0.031	0.022	0.060	0.058	0.0557	0.054
**H3av**	0.001	0.028	0.038	0.029	0.026	0.039	0.042	0.040
**H7av**	-0.146	-0.096	-0.075	-0.083	-0.100	-0.055	-0.038	-0.040
**H8**	-0.428	-0.181	-0.076	-0.079	-0.200	0.044	0.145	0.149
RMS	**0.226**	**0.104**	**0.059**	**0.060**	**0.117**	**0.050**	**0.083**	**0.084**
**N1**	27.929	19.668	16.437	16.265	-12.802	-21.070	-24.201	-24.357
**N3**	26.339	19.897	17.496	17.368	-15.655	-22.139	-24.463	-24.574
**N7**	19.855	14.688	12.712	12.604	-12.064	-17.768	-19.931	-20.038
**N9**	20.120	6.729	1.503	1.228	-7.034	-21.667	-27.329	-27.614
RMS	**23.837**	**16.153**	**13.603**	**13.477**	**12.288**	**20.732**	**24.126**	**24.296**
**O2**	37.013	25.079	19.409	19.095	33.858	19.778	13.279	12.924
**O6**	15.523	5.225	0.144	-0.138	13.015	1.438	-4.182	-4.492
RMS	**28.381**	**18.114**	**13.725**	**13.503**	**25.649**	**14.022**	**9.844**	**9.675**

[a] experimental data for ^1H, ^{13}C and ^{15}N NMR are from[1], ^{17}O NMR from this work

[b] methyl protons shieldings are averaged;

[c] the RMS deviations are shown.

Table S4. Cont.

Nuclei	LC-BLYP				M06			
	**Gas**	**$CHCl_3$**	**DMSO**	**H_2O**	**Gas**	**$CHCl_3$**	**DMSO**	**H_2O**
**C1**	-0.605	-10.130	0.145	-7.530	0.334	2.070	-0.867	0.914
**C2**	13.998	2.853	16.631	5.958	6.524	8.672	7.158	8.968
**C3**	-0.346	-10.136	0.526	-7.503	3.116	3.647	2.293	4.081
**C4**	14.488	-0.817	15.530	1.852	10.207	11.548	9.246	11.031
**C5**	8.305	-5.067	9.219	-2.403	6.351	6.046	5.596	7.387
**C6**	15.942	3.379	18.240	6.384	5.605	8.788	5.484	7.286
**C7**	1.201	-8.568	2.165	-5.930	3.683	4.819	2.790	4.575
**C8**	11.815	0.988	16.071	4.594	7.554	11.309	9.924	11.758
RMS	**10.431**	**6.380**	**12.241**	**5.643**	**6.113**	**7.835**	**6.229**	**7.798**
**H1av**	0.074	-0.093	0.068	0.256	-0.02743	-0.021	-0.038	-0.056
**H3av**	0.026	-0.143	0.037	0.213	0.0336	-0.008	0.049	0.031
**H7av**	-0.098	-0.209	-0.036	0.162	-0.10317	-0.058	-0.048	-0.065
**H8**	-0.116	-0.033	0.242	0.433	-0.1247	0.103	0.224	0.212
RMS	**0.085**	**0.136**	**0.128**	**0.285**	**0.084**	**0.060**	**0.119**	**0.115**
**N1**	-24.166	-44.037	-35.182	-47.221	-11.534	-20.328	-23.855	-24.028
**N3**	-27.967	-44.485	-36.493	-46.880	-13.999	-20.723	-23.804	-23.935
**N7**	-19.942	-39.357	-27.580	-41.618	-9.574	-15.345	-18.021	-18.140
**N9**	-13.512	-45.876	-34.117	-51.760	-0.371	-15.719	-21.060	-21.356
RMS	**22.059**	**43.508**	**33.519**	**47.007**	**10.257**	**18.202**	**21.817**	**21.996**
**O2**	31.265	-4.859	9.499	-11.801	38.317	23.993	18.947	18.607
**O6**	11.691	-21.545	-5.566	-27.208	8.316	6.837	-9.231	-9.520
RMS	**23.603**	**15.617**	**7.785**	**20.971**	**27.616**	**18.756**	**15.124**	**15.564**
Nuclei	**PBE0**				**TPSSh**			
**C1**	0.581	0.921	1.261	1.283	1.804	2.206	2.379	2.377
**C2**	5.334	6.776	7.559	7.604	1.716	3.083	3.645	3.663
**C3**	1.445	1.952	2.349	2.374	2.351	2.904	3.125	3.125
**C4**	7.109	7.618	8.015	8.041	2.623	3.166	3.382	3.382
**C5**	6.285	6.792	7.240	7.269	3.425	4.046	4.353	4.359
**C6**	5.693	6.842	7.525	7.566	1.063	2.125	2.580	2.593
**C7**	3.045	3.631	4.061	4.088	4.100	4.841	5.142	5.147
**C8**	3.344	6.146	7.473	7.547	-1.436	1.336	2.459	2.506
RMS	**4.661**	**5.618**	**6.224**	**6.260**	**2.508**	**3.141**	**3.504**	**3.514**
**H1av**	-0.712	-0.001	-0.005	-0.005	0.032	0.030	0.033	0.032
**H3av**	-0.018	-0.001	0.002	0.002	0.002	0.017	0.027	0.026
**H7av**	-0.138	-0.092	-0.077	-0.076	-0.144	-0.101	-0.078	-0.078
**H8**	-0.229	0.016	0.116	0.122	-0.305	-0.063	0.043	0.048
RMS	**0.381**	**0.047**	**0.070**	**0.072**	**0.170**	**0.062**	**0.049**	**0.050**
**N1**	6.479	-1.438	-4.463	-4.617	12.379	4.479	1.433	1.277
**N3**	4.612	-1.531	-3.752	-3.863	12.752	6.626	4.384	4.271
**N7**	4.264	-0.915	-2.883	-2.984	8.604	3.471	1.507	1.407
**N9**	7.832	-5.836	-11.103	-11.372	7.450	-5.758	-10.884	-11.146
RMS	**5.974**	**3.135**	**6.434**	**6.604**	**10.552**	**5.224**	**5.958**	**6.043**
**O2**	35.085	22.112	16.133	15.806	22.308	10.400	4.808	4.501
**O6**	16.318	5.040	-0.387	-0.685	1.443	-8.855	-13.890	-14.169
RMS	**24.921**	**16.228**	**12.572**	**12.385**	**15.807**	**9.658**	**10.394**	**10.512**
Nuclei	**wB97X**							
**C1**	0.295	0.608	0.994	1.012				
**C2**	7.398	8.939	9.815	9.858				
**C3**	0.683	1.127	1.554	1.574				
**C4**	8.213	8.710	9.149	9.170				
**C5**	5.000	5.394	5.853	5.876				
**C6**	8.666	9.883	10.673	10.714				
**C7**	2.276	2.837	3.298	3.319				
**C8**	4.948	7.701	9.039	9.105				
RMS	**5.618**	**6.629**	**7.282**	**7.314**				
**H1av**	0.008	0.004	0.004	-0.001				
**H3av**	-0.018	-0.009	-0.004	-0.008				
**H7av**	-0.142	-0.102	-0.084	-0.087				
**H8**	-0.213	0.0297	0.1325	0.133				
RMS	**0.128**	**0.053**	**0.079**	**0.080**				
**N1**	-9.465	-17.412	-20.415	-20.568				
**N3**	-11.581	-17.865	-20.115	-20.227				
**N7**	-7.714	-13.224	-15.312	-15.419				
**N9**	-5.791	-20.195	-25.773	-26.059				
RMS	**8.898**	**17.357**	**20.737**	**20.910**				
**O2**	27.429	13.724	7.405	7.059				
**O6**	7.288	-4.287	-9.696	-9.984				
RMS	**20.068**	**10.166**	**8.627**	**8.646**				

[a] the RMS deviations are shown

Table S5. Indirect spin-spin coupling constants (in Hz) of caffeine calculated with selected functionals combined with "mixed" aug-cc-pVTZ basis set[a]

$^{n}J_{X\text{-}Y}$	B3LYP				BLYP			
	Gas	$CHCl_3$	DMSO	H_2O	Gas	$CHCl_3$	DMSO	H_2O
**C8-H8**	215.837	219.087	220.465	220.537	222.002	225.280	226.694	226.768
**N7-H8**	6.132	5.941	5.856	5.852	6.886	6.703	6.622	6.127
**C7-H8**	1.763	1.836	1.857	1.858	1.631	1.710	1.731	1.732
**C8-H7**	4.326	4.414	4.438	4.439	4.276	4.370	4.394	4.395
**N9-H8**	10.003	10.060	10.082	10.083	10.603	10.681	10.711	10.713
**C4-H8**	13.395	13.467	13.497	13.499	13.661	13.780	13.831	13.833
**N3-H8**	0.207	0.229	0.238	0.238	0.216	0.243	0.255	0.255
**C7-H7**	144.954	145.871	146.155	146.168	147.221	148.205	148.515	148.530
**N7-H7**	-0.689	-0.702	-0.712	-0.713	-0.202	-0.212	-0.221	-0.255
**H8-H7**	-0.742	-0.776	-0.789	-0.790	-0.649	-0.682	-0.694	-0.695
**N9-H7**	0.008	0.002	-0.001	-0.001	0.014	0.012	0.011	0.011
**C5-H7**	2.256	2.386	2.436	2.438	2.203	2.347	2.403	2.406
**C6-H7**	-0.311	-0.245	-0.249	-0.249	-0.229	-0.233	-0.236	-0.236
**C3-H3**	145.238	145.777	145.945	145.952	147.608	148.202	148.386	148.394
**N3-H3**	-0.479	-0.475	-0.475	-0.475	-0.070	-0.063	-0.062	-0.062
**C2-H3**	3.165	3.223	3.245	3.246	3.249	3.304	3.324	3.326
**N1-H3**	-0.083	-0.087	-0.088	-0.088	-0.062	-0.064	-0.064	-0.064
**C4-H3**	2.792	2.811	2.819	2.820	2.816	2.831	2.837	2.838
**C5-H3**	0.122	0.101	0.092	0.092	0.141	0.125	0.119	0.118
**N9-H3**	-0.077	-0.077	-0.078	-0.0782	-0.048	-0.046	-0.047	-0.047
**C1-H1**	145.153	145.404	145.465	145.467	147.371	147.646	147.710	147.712
**N1-H1**	-0.369	-0.362	-0.362	-0.362	0.057	0.064	0.064	0.064
**C2-H1**	3.105	3.118	3.122	3.123	3.228	3.232	3.232	3.232
**C6-H1**	2.632	2.647	2.650	2.650	2.667	2.678	2.678	2.678
**C5-H1**	-0.161	-0.170	-0.175	-0.176	-0.083	-0.092	-0.096	-0.096
**N3-H1**	-0.095	-0.098	-0.100	-0.100	-0.075	-0.077	-0.078	-0.078
**C6-H8**	1.187	1.185	1.182	1.182	1.102	1.121	1.128	1.128
**C5-H8**	4.477	4.803	4.934	4.941	4.592	4.911	5.040	5.047
$^{n}J_{X\text{-}Y}$	**BP86**				**CAM-B3LYP**			
**C8-H8**	200.470	203.648	205.022	205.094	211.033	214.327	215.710	215.780
**N7-H8**	6.380	6.221	6.150	6.146	5.867	5.671	5.584	5.579
**C7-H8**	1.503	1.569	1.585	1.586	1.945	2.016	2.036	2.038
**C8-H7**	3.933	4.023	4.046	4.047	4.282	4.365	4.387	4.388
**N9-H8**	9.861	9.937	9.966	9.968	9.996	10.038	10.053	10.054
**C4-H8**	13.012	13.129	13.177	13.180	13.242	13.281	13.297	13.298
**N3-H8**	0.206	0.233	0.244	0.245	0.185	0.205	0.213	0.213
**C7-H7**	131.340	132.252	132.548	132.562	141.808	142.694	142.967	142.979
**N7-H7**	-0.370	-0.383	-0.393	-0.394	-0.834	-0.845	-0.855	-0.856
**H8-H7**	-0.678	-0.708	-0.719	-0.719	-0.768	-0.803	-0.816	-0.817
**N9-H7**	-0.020	-0.021	-0.021	-0.021	-0.013	-0.019	-0.022	-0.022
**C5-H7**	2.103	2.245	2.299	2.302	2.251	2.371	2.415	2.418
**C6-H7**	-0.221	-0.225	-0.228	-0.228	-0.239	-0.244	-0.248	-0.248
**C3-H3**	131.673	132.223	132.401	132.409	141.930	142.431	142.586	142.593
**N3-H3**	-0.229	-0.223	-0.221	-0.221	-0.596	-0.591	-0.591	-0.591
**C2-H3**	3.101	3.154	3.174	3.175	3.060	3.115	3.137	3.1373
**N1-H3**	-0.071	-0.072	-0.073	-0.073	-0.092	-0.095	-0.097	-0.097
**C4-H3**	2.736	2.753	2.759	2.759	2.750	2.770	2.778	2.778
**C5-H3**	0.122	0.111	0.106	0.105	0.121	0.100	0.090	0.090
**N9-H3**	-0.120	-0.119	-0.120	-0.121	-0.085	-0.086	-0.088	-0.088
**C1-H1**	131.465	131.719	131.788	131.791	141.942	142.189	142.250	142.252
**N1-H1**	-0.073	-0.067	-0.067	-0.067	-0.490	-0.483	-0.483	-0.483
**C2-H1**	3.087	3.092	3.091	3.091	2.973	2.990	2.996	2.996
**C6-H1**	2.540	2.552	2.552	2.552	2.568	2.582	2.587	2.587
**C5-H1**	-0.094	-0.101	-0.105	-0.105	-0.189	-0.198	-0.203	-0.203
**N3-H1**	-0.086	-0.087	-0.089	-0.089	-0.101	-0.105	-0.106	-0.106
**C6-H8**	1.072	1.093	1.100	1.100	1.215	1.207	1.199	1.197
**C5-H8**	4.405	4.701	4.823	4.829	4.426	4.745	4.871	4.877

[a] methyl couplings are averaged.

Table S5. Cont.

$^{n}J_{X\text{-}Y}$	LC-BLYP				M06			
	**Gas**	**$CHCl_3$**	**DMSO**	**H_2O**	**Gas**	**$CHCl_3$**	**DMSO**	**H_2O**
**C8-H8**	202.763	206.967	207.445	210.359	239.840	246.098	248.771	248.905
**N7-H8**	5.583	5.362	5.307	5.349	5.0787	4.952	4.882	4.879
**C7-H8**	2.222	2.209	2.305	2.271	2.114	2.168	2.181	2.184
**C8-H7**	4.151	4.167	4.242	4.275	4.987	5.058	5.073	5.075
**N9-H8**	9.968	9.847	9.984	10.032	8.765	8.960	9.050	9.053
**C4-H8**	12.786	12.693	12.792	12.930	13.964	14.133	14.222	14.225
**N3-H8**	0.132	0.146	0.155	0.173	0.223	0.241	0.248	0.249
**C7-H7**	135.447	136.880	136.510	138.297	157.332	158.635	159.079	159.096
**N7-H7**	-0.849	-0.863	-0.862	-0.875	-1.4981	-1.547	-1.570	-1.571
**H8-H7**	-0.780	-0.838	-0.828	-0.854	-1.097	-1.117	-1.123	-1.123
**N9-H7**	-0.051	-0.080	-0.060	-0.057	0.054	0.043	0.038	0.038
**C5-H7**	2.239	2.324	2.384	2.418	2.481	2.641	2.687	2.692
**C6-H7**	-0.243	-0.229	-0.250	-0.242	-0.197	-0.196	-0.208	-0.211
**C3-H3**	135.380	136.386	135.953	137.687	157.769	158.435	158.630	158.638
**N3-H3**	-0.618	-0.612	-0.608	-0.612	-1.220	-1.225	-1.230	-1.230
**C2-H3**	2.936	2.976	3.002	3.042	3.495	3.549	3.570	3.571
**N1-H3**	-0.087	-0.103	-0.093	-0.094	-0.104	-0.106	-0.108	-0.108
**C4-H3**	2.727	2.730	2.753	2.780	3.111	3.124	3.129	3.129
**C5-H3**	0.122	0.065	0.095	0.097	0.047	0.023	0.011	0.010
**N9-H3**	-0.097	-0.104	-0.100	-0.116	-0.226	-0.226	-0.227	-0.227
**C1-H1**	135.488	136.271	135.776	137.491	157.648	157.952	157.983	157.982
**N1-H1**	-0.512	-0.508	-0.504	-0.503	-1.077	-1.0812	-1.079	-1.079
**C2-H1**	2.837	2.856	2.860	2.902	3.413	3.414	3.417	3.418
**C6-H1**	2.496	2.510	2.512	2.546	2.881	2.896	2.896	2.894
**C5-H1**	-0.185	-0.230	-0.198	-0.199	-0.340	-0.347	-0.349	-0.347
**N3-H1**	-0.093	-0.109	-0.097	-0.099	-0.107	-0.110	-0.112	-0.112
**C6-H8**	1.200	1.154	1.174	1.230	1.195	1.172	1.163	1.166
**C5-H8**	4.337	4.481	4.745	4.801	4.093	4.538	4.720	4.725
$^{n}J_{X\text{-}Y}$	**PBE0**				**TPSSh**			
**C8-H8**	202.569	205.811	207.183	207.254	228.305	232.299	234.002	234.092
**N7-H8**	5.563	5.380	5.299	5.295	6.721	6.529	6.445	6.441
**C7-H8**	1.739	1.807	1.827	1.828	1.444	1.522	1.544	1.545
**C8-H7**	4.312	4.410	4.438	4.439	4.383	4.488	4.518	4.520
**N9-H8**	9.387	9.443	9.464	9.465	10.453	10.506	10.526	10.527
**C4-H8**	13.392	13.453	13.479	13.481	14.456	14.532	14.565	14.566
**N3-H8**	0.216	0.237	0.246	0.246	0.271	0.298	0.309	0.310
**C7-H7**	135.741	136.603	136.877	136.889	156.795	157.940	158.307	158.324
**N7-H7**	-1.107	-1.130	-1.143	-1.144	-0.627	-0.647	-0.660	-0.661
**H8-H7**	-0.843	-0.881	-0.895	-0.896	-0.780	-0.820	-0.835	-0.835
**N9-H7**	-0.012	-0.019	-0.023	-0.023	0.022	0.015	0.012	0.012
**C5-H7**	2.285	2.417	2.467	2.470	2.267	2.409	2.463	2.466
**C6-H7**	-0.235	-0.242	-0.247	-0.247	-0.234	-0.243	-0.248	-0.248
**C3-H3**	135.890	136.386	136.546	136.552	157.087	157.733	157.935	157.944
**N3-H3**	-0.848	-0.847	-0.848	-0.848	-0.428	-0.424	-0.424	-0.424
**C2-H3**	3.205	3.269	3.295	3.296	3.349	3.418	3.445	3.446
**N1-H3**	-0.103	-0.107	-0.109	-0.109	-0.085	-0.088	-0.089	-0.089
**C4-H3**	2.848	2.872	2.881	2.881	2.876	2.898	2.907	2.907
**C5-H3**	0.103	0.081	0.071	0.070	0.122	0.098	0.087	0.087
**N9-H3**	-0.124	-0.127	-0.129	-0.129	-0.096	-0.098	0.296	-0.100
**C1-H1**	135.803	136.030	136.089	136.091	156.909	157.193	157.262	157.264
**N1-H1**	-0.713	-0.707	-0.706	-0.706	-0.286	-0.277	-0.276	-0.276
**C2-H1**	3.151	3.169	3.176	3.176	3.302	3.319	3.325	3.325
**C6-H1**	2.682	2.700	2.705	2.705	2.745	2.763	2.767	2.767
**C5-H1**	-0.214	-0.222	-0.227	-0.227	-0.196	-0.204	-0.209	-0.209
**N3-H1**	-0.117	-0.120	-0.122	-0.122	-0.104	-0.107	-0.109	-0.109
**C6-H8**	1.272	1.260	1.253	1.253	1.344	1.347	1.347	1.347
**C5-H8**	4.390	4.720	4.852	4.859	4.818	5.176	5.320	5.327

Table S5. Cont.

$^{n}J_{X\text{-}Y}$	wB97X			
	**Gas**	**$CHCl_3$**	**DMSO**	**H_2O**
**C8-H8**	190.617	193.905	195.277	195.343
**N7-H8**	5.163	4.983	4.901	4.887
**C7-H8**	1.895	1.953	1.972	1.959
**C8-H7**	4.029	4.117	4.136	4.136
**N9-H8**	9.176	9.216	9.230	9.236
**C4-H8**	12.380	12.411	12.426	12.417
**N3-H8**	0.179	0.197	0.202	0.206
**C7-H7**	126.531	127.357	127.628	127.636
**N7-H7**	-1.167	-1.188	-1.201	-1.204
**H8-H7**	-0.648	-0.685	-0.700	-0.701
**N9-H7**	-0.013	-0.021	-0.025	-0.023
**C5-H7**	2.060	2.168	2.208	2.209
**C6-H7**	-0.247	-0.254	-0.257	-0.260
**C3-H3**	126.506	126.965	127.108	127.117
**N3-H3**	-0.934	-0.933	-0.933	-0.933
**C2-H3**	2.829	2.887	2.910	2.912
**N1-H3**	-0.101	-0.105	-0.107	-0.112
**C4-H3**	2.533	2.551	2.559	2.558
**C5-H3**	0.084	0.063	0.055	0.052
**N9-H3**	-0.023	-0.021	-0.022	-0.021
**C1-H1**	126.502	126.742	126.805	126.807
**N1-H1**	-0.832	-0.827	-0.828	-0.826
**C2-H1**	2.735	2.757	2.765	2.765
**C6-H1**	2.377	2.394	2.399	2.400
**C5-H1**	-0.232	-0.240	-0.244	-0.244
**N3-H1**	-0.108	-0.111	-0.113	-0.113
**C6-H8**	1.166	1.474	1.143	1.136
**C5-H8**	4.142	4.439	4.555	4.559

Table S6. Deviations of theoretical spin-spin coupling constants from available experimental values (in Hz) of caffeine calculated with selected density functionals combined with "mixed" aug-cc-pVTZ basis set[a]

$^nJ_{X-Y}$	**B3LYP**				**BLYP**				**Exp.**
	**Gas**	**CHCl₃**	**DMSO**	**H₂O**	**Gas**	**CHCl₃**	**DMSO**	**H₂O**	
**H8-H7**	-0.042	-0.076	-0.089	-0.090	0.051	0.018	0.006	0.006	**0.7**
**C2-H1**	0.405	0.418	0.422	0.423	0.528	0.532	0.532	0.532	**2.7**
**C2-H3**	0.265	0.323	0.345	0.346	0.349	0.404	0.424	0.426	**2.9**
**C4-H8**	0.695	0.767	0.797	0.799	0.961	1.080	1.131	1.133	**12.7**
**C4-H3**	-0.008	0.011	0.019	0.020	0.016	0.031	0.037	0.038	**2.8**
**C6-H8**	0.187	0.185	0.182	0.182	0.102	0.121	0.128	0.128	**1**
**C6-H1**	0.032	0.047	0.050	0.050	0.067	0.078	0.078	0.078	**2.6**
**C5-H8**	-0.323	0.003	0.134	0.141	-0.209	0.111	0.240	0.247	**4.8**
**C5-H7**	-0.144	-0.014	0.036	0.038	-0.197	-0.053	0.003	0.006	**2.4**
**C8-H8**	6.037	9.287	10.665	10.737	12.202	15.480	16.894	16.968	**209.8**
**C8-H7**	0.226	0.314	0.338	0.339	0.176	0.270	0.294	0.295	**4.1**
**C1-H1**	3.453	3.704	3.765	3.767	5.671	5.946	6.010	6.012	**141.7**
**C3-H3**	3.338	3.877	4.045	4.052	5.708	6.302	6.486	6.494	**141.9**
**C7-H7**	2.754	3.671	3.955	3.968	5.021	6.005	6.315	6.330	**142.2**
**C7-H8**	0.363	0.436	0.457	0.458	0.231	0.310	0.331	0.332	**1.4**
**N1-H1**	0.831	0.838	0.839	0.839	1.257	1.264	1.264	1.264	**1.2**
**N3-H3**	0.921	0.926	0.926	0.926	1.330	1.337	1.338	1.338	**1.4**
**N7-H8**	0.132	-0.059	-0.144	-0.148	0.886	0.703	0.622	0.127	**6**
**N7-H7**	-2.489	-2.502	-2.512	-2.513	-2.002	-2.012	-2.021	-2.055	**1.8**
**N9-H8**	-2.097	-2.040	-2.018	-2.017	-1.497	-1.419	-1.389	-1.388	**12.1**
$^nJ_{X-Y}$	**BP86**				**CAM-B3LYP**				**Exp.**
**H8-H7**	0.022	-0.008	-0.019	-0.019	-0.068	-0.1023	-0.116	-0.117	**0.7**
**C2-H1**	0.387	0.392	0.391	0.391	0.273	0.290	0.296	0.296	**2.7**
**C2-H3**	0.201	0.254	0.274	0.275	0.160	0.215	0.237	0.237	**2.9**
**C4-H8**	0.312	0.429	0.477	0.480	0.542	0.581	0.597	0.598	**12.7**
**C4-H3**	-0.065	-0.047	-0.041	-0.041	-0.050	-0.030	-0.022	-0.022	**2.8**
**C6-H8**	0.072	0.093	0.100	0.100	0.215	0.207	0.199	0.197	**1**
**C6-H1**	-0.060	-0.049	-0.048	-0.048	-0.032	-0.012	-0.013	-0.013	**2.6**
**C5-H8**	-0.395	-0.099	0.023	0.029	-0.374	-0.055	0.071	0.077	**4.8**
**C5-H7**	-0.298	-0.156	-0.101	-0.098	-0.149	-0.029	0.015	0.018	**2.4**
**C8-H8**	-9.330	-6.152	-4.778	-4.706	1.233	4.527	5.910	5.980	**209.8**
**C8-H7**	-0.167	-0.077	-0.054	-0.053	0.182	0.265	0.287	0.288	**4.1**
**C1-H1**	-10.235	-9.981	-9.912	-9.909	0.242	0.489	0.550	0.552	**141.7**
**C3-H3**	-10.227	-9.677	-9.499	-9.491	0.030	0.531	0.686	0.693	**141.9**
**C7-H7**	-10.860	-9.948	-9.652	-9.638	-0.392	0.494	0.767	0.779	**142.2**
**C7-H8**	0.103	0.169	0.185	0.186	0.545	0.616	0.636	0.638	**1.4**
**N1-H1**	1.127	1.133	1.133	1.133	0.710	0.717	0.717	0.717	**1.2**
**N3-H3**	1.171	1.177	1.179	1.179	0.804	0.809	0.809	0.809	**1.4**
**N7-H8**	0.380	0.221	0.150	0.146	-0.133	-0.330	-0.417	-0.421	**6**
**N7-H7**	-2.170	-2.183	-2.193	-2.194	-2.634	-2.645	-2.655	-2.656	**1.8**
**N9-H8**	-2.239	-2.163	-2.134	-2.133	-2.104	-2.062	-2.047	-2.046	**12.1**
$^nJ_{X-Y}$	**LC-BLYP**				**M06**				**Exp.**
**H8-H7**	-0.080	-0.138	-0.128	-0.154	-0.397	-0.417	-0.423	-0.423	**0.7**
**C2-H1**	0.137	0.156	0.160	0.202	0.713	0.714	0.717	0.718	**2.7**
**C2-H3**	0.036	0.076	0.102	0.142	0.595	0.649	0.670	0.671	**2.9**
**C4-H8**	0.086	-0.007	0.092	0.230	1.264	1.433	1.522	1.525	**12.7**
**C4-H3**	-0.073	-0.070	-0.047	-0.021	0.311	0.324	0.329	0.329	**2.8**
**C6-H8**	0.200	0.154	0.174	0.230	0.195	0.172	0.163	0.166	**1**
**C6-H1**	-0.104	-0.090	-0.088	-0.054	0.281	0.296	0.296	0.294	**2.6**
**C5-H8**	-0.463	-0.319	-0.055	0.001	-0.707	-0.262	-0.080	-0.075	**4.8**
**C5-H7**	-0.161	-0.076	-0.016	0.018	0.081	0.241	0.287	0.292	**2.4**
**C8-H8**	-7.037	-2.833	-2.355	0.559	30.040	36.298	38.971	39.105	**209.8**
**C8-H7**	0.051	0.070	0.142	0.175	0.887	0.958	0.973	0.975	**4.1**
**C1-H1**	-6.212	-5.429	-5.924	-4.209	15.948	16.252	16.283	16.282	**141.7**
**C3-H3**	-6.520	-5.514	-5.947	-4.213	15.869	16.535	16.730	16.738	**141.9**
**C7-H7**	-6.753	-5.320	-5.690	-3.903	15.132	16.435	16.879	16.896	**142.2**
**C7-H8**	0.822	0.809	0.905	0.871	0.714	0.768	0.781	0.784	**1.4**
**N1-H1**	0.688	0.692	0.696	0.697	0.123	0.119	0.121	0.121	**1.2**
**N3-H3**	0.782	0.788	0.792	0.788	0.180	0.176	0.170	0.170	**1.4**
**N7-H8**	-0.417	-0.638	-0.693	-0.651	-0.921	-1.048	-1.118	-1.121	**6**
**N7-H7**	-2.649	-2.663	-2.662	-2.675	-3.298	-3.347	-3.370	-3.371	**1.8**
**N9-H8**	-2.132	-2.253	-2.116	-2.068	-3.335	-3.140	-3.051	-3.047	**12.1**

[a] methyl couplings are averaged.

Table S6. Cont.

$^{n}J_{X\text{-}Y}$	PBE0				TPSSh				Exp.
	**Gas**	**$CHCl_3$**	**DMSO**	**H_2O**	**Gas**	**$CHCl_3$**	**DMSO**	**H_2O**	
**H8-H7**	-0.143	-0.181	-0.195	-0.196	-0.080	-0.120	-0.135	-0.135	**0.7**
**C2-H1**	0.451	0.469	0.476	0.476	0.602	0.619	0.625	0.625	**2.7**
**C2-H3**	0.305	0.369	0.395	0.396	0.449	0.518	0.545	0.546	**2.9**
**C4-H8**	0.692	0.753	0.779	0.781	1.756	1.832	1.865	1.866	**12.7**
**C4-H3**	0.048	0.072	0.081	0.081	0.076	0.098	0.107	0.107	**2.8**
**C6-H8**	0.272	0.260	0.253	0.253	0.344	0.347	0.347	0.347	**1**
**C6-H1**	0.082	0.100	0.105	0.105	0.145	0.163	0.167	0.167	**2.6**
**C5-H8**	-0.411	-0.080	0.052	0.059	0.018	0.376	0.520	0.527	**4.8**
**C5-H7**	-0.115	0.017	0.067	0.070	-0.133	0.009	0.063	0.066	**2.4**
**C8-H8**	-7.231	-3.989	-2.617	-2.546	18.505	22.499	24.202	24.292	**209.8**
**C8-H7**	0.212	0.310	0.338	0.339	0.283	0.388	0.418	0.420	**4.1**
**C1-H1**	-5.897	-5.670	-5.611	-5.609	15.209	15.493	15.562	15.564	**141.7**
**C3-H3**	-6.010	-5.514	-5.354	-5.348	15.187	15.833	16.035	16.044	**141.9**
**C7-H7**	-6.459	-5.597	-5.323	-5.311	14.595	15.740	16.107	16.124	**142.2**
**C7-H8**	0.339	0.407	0.427	0.428	0.044	0.122	0.144	0.145	**1.4**
**N1-H1**	0.487	0.494	0.494	0.494	0.914	0.923	0.924	0.924	**1.2**
**N3-H3**	0.552	0.553	0.552	0.552	0.972	0.976	0.977	0.977	**1.4**
**N7-H8**	-0.437	-0.620	-0.701	-0.705	0.721	0.529	0.445	0.441	**6**
**N7-H7**	-2.907	-2.930	-2.943	-2.944	-2.427	-2.447	-2.460	-2.461	**1.8**
**N9-H8**	-2.713	-2.657	-2.636	-2.635	-1.647	-1.594	-1.574	-1.573	**12.1**

$^{n}J_{X\text{-}Y}$	wB97X				Exp.
**H8-H7**	0.052	0.015	0.001	-0.001	**0.7**
**C2-H1**	0.035	0.057	0.065	0.065	**2.7**
**C2-H3**	-0.071	-0.013	0.010	0.012	**2.9**
**C4-H8**	-0.320	-0.289	-0.274	-0.283	**12.7**
**C4-H3**	-0.267	-0.249	-0.242	-0.242	**2.8**
**C6-H8**	0.166	0.474	0.143	0.136	**1**
**C6-H1**	-0.223	-0.206	-0.201	-0.200	**2.6**
**C5-H8**	-0.658	-0.361	-0.245	-0.241	**4.8**
**C5-H7**	-0.340	-0.233	-0.192	-0.191	**2.4**
**C8-H8**	-19.183	-15.895	-14.523	-14.457	**209.8**
**C8-H7**	-0.071	0.017	0.036	0.036	**4.1**
**C1-H1**	-15.198	-14.958	-14.895	-14.893	**141.7**
**C3-H3**	-15.394	-14.935	-14.792	-14.783	**141.9**
**C7-H7**	-15.669	-14.843	-14.572	-14.564	**142.2**
**C7-H8**	0.495	0.553	0.572	0.559	**1.4**
**N1-H1**	0.368	0.373	0.372	0.374	**1.2**
**N3-H3**	0.466	0.467	0.467	0.467	**1.4**
**N7-H8**	-0.837	-1.017	-1.099	-1.113	**6**
**N7-H7**	-2.967	-2.988	-3.001	-3.004	**1.8**
**N9-H8**	-2.924	-2.885	-2.870	-2.864	**12.1**

Table S7. Deviations of selected $^{1}J_{C\text{-}H}$ theoretical spin-spin coupling constants from available experimental values[a] (in Hz) of caffeine calculated with selected density functionals combined with “mixed” aug-cc-pVTZ basis set[b]

$^{1}J_{C\text{-}H}$	**B3LYP**				**BLYP**				**Exp.**
	**Gas**	**$CHCl_3$**	**DMSO**	**H_2O**	**Gas**	**$CHCl_3$**	**DMSO**	**H_2O**	
**C8-H8**	6.037	9.287	10.665	10.737	12.202	15.480	16.894	16.968	**209.8**
**C1-H1**	3.453	3.704	3.765	3.767	5.671	5.946	6.010	6.012	**141.7**
**C3-H3**	3.338	3.877	4.045	4.052	5.708	6.302	6.486	6.494	**141.9**
**C7-H7**	2.754	3.671	3.955	3.968	5.021	6.005	6.315	6.330	**142.2**
**RMS**	**4.096**	**5.667**	**6.323**	**6.357**	**7.727**	**9.364**	**10.043**	**10.078**	-
$^{1}J_{C\text{-}H}$	**BP86**				**CAM-B3LYP**				**Exp.**
**C8-H8**	-9.330	-6.152	-4.778	-4.706	1.233	4.527	5.910	5.980	**209.8**
**C1-H1**	-10.235	-9.981	-9.912	-9.909	0.242	0.489	0.550	0.552	**141.7**
**C3-H3**	-10.227	-9.677	-9.499	-9.491	0.030	0.531	0.686	0.693	**141.9**
**C7-H7**	-10.860	-9.948	-9.652	-9.638	-0.392	0.494	0.767	0.779	**142.2**
**RMS**	**10.178**	**9.084**	**8.724**	**8.708**	**0.658**	**2.305**	**3.012**	**3.048**	-
$^{1}J_{C\text{-}H}$	**LC-BLYP**				**M06**				**Exp.**
**C8-H8**	-7.037	-2.833	-2.355	0.559	30.040	36.298	38.971	39.105	**209.8**
**C1-H1**	-6.212	-5.429	-5.924	-4.209	15.948	16.252	16.283	16.282	**141.7**
**C3-H3**	-6.520	-5.514	-5.947	-4.213	15.869	16.535	16.730	16.738	**141.9**
**C7-H7**	-6.753	-5.320	-5.690	-3.903	15.132	16.435	16.879	16.896	**142.2**
**RMS**	**6.637**	**4.904**	**5.206**	**3.571**	**20.233**	**23.050**	**24.232**	**24.290**	-
$^{1}J_{C\text{-}H}$	**PBE0**				**TPSSh**				**Exp.**
**C8-H8**	-7.231	-3.989	-2.617	-2.546	18.505	22.499	24.202	24.292	**209.8**
**C1-H1**	-5.897	-5.670	-5.611	-5.609	15.209	15.493	15.562	15.564	**141.7**
**C3-H3**	-6.010	-5.514	-5.354	-5.348	15.187	15.833	16.035	16.044	**141.9**
**C7-H7**	-6.459	-5.597	-5.323	-5.311	14.595	15.740	16.107	16.124	**142.2**
**RMS**	**6.421**	**5.239**	**4.882**	**4.867**	**15.948**	**17.640**	**18.334**	**18.369**	-
$^{1}J_{C\text{-}H}$	**wB97X**				**Exp.**				
**C8-H8**	-19.183	-15.895	-14.523	-14.457	**209.8**				
**C1-H1**	-15.198	-14.958	-14.895	-14.893	**141.7**				
**C3-H3**	-15.394	-14.935	-14.792	-14.783	**141.9**				
**C7-H7**	-15.669	-14.843	-14.572	-14.564	**142.2**				
**RMS**	**16.443**	**15.164**	**14.696**	**14.675**	-				

[a] experimental data from[1]

[b] methyl SSCC are averaged.

Appendix I. Cartesian cordinates of caffeine calculated at **B3LYP/aug-cc-pVTZ** level of theory in vacuum and solvents in PCM model

Vacuum

0 1

C	2.44333100	1.36790400	-0.00001100
H	3.42345300	1.81596700	-0.00001400
C	0.90487400	-0.17682600	-0.00000200
C	0.13314400	-1.37891400	0.00000900
O	0.57177900	-2.52050500	0.00002200
N	-1.25238600	-1.09967400	0.00000400
C	-1.85046900	0.16977400	0.00000100
C	0.35084900	1.08402000	-0.00000900
N	2.27427000	0.02839900	-0.00000300
N	1.30410400	2.04690800	-0.00001500
N	-1.00748100	1.27020400	-0.00000800
C	-1.60660400	2.60263800	-0.00000800
H	-2.22973000	2.73236700	0.88257100
H	-0.80073400	3.32854900	-0.00004300
H	-2.22978500	2.73234000	-0.88255100
O	-3.06135800	0.29816200	0.00000500
C	-2.18455200	-2.22987200	0.00000900
H	-2.82036300	-2.18804100	-0.88166000
H	-1.59469700	-3.13882100	-0.00003500
H	-2.82029900	-2.18809200	0.88172800
C	3.31402800	-0.98971200	0.00000000
H	3.22564200	-1.61948200	-0.88215800
H	4.28039600	-0.49145400	0.00002800
H	3.22560500	-1.61951200	0.88213300

$CHCl_3$

0 1

C	2.44646400	1.36000000	-0.00000800
H	3.42708500	1.80621200	-0.00001000
C	0.90584400	-0.18161400	-0.00000200
C	0.12857700	-1.37673900	0.00000500
O	0.55830300	-2.52540800	0.00001200
N	-1.25640800	-1.09557900	0.00000400
C	-1.84725700	0.17349800	0.00000200
C	0.35192600	1.08053800	-0.00000700
N	2.27585300	0.02396300	-0.00000300
N	1.30542700	2.04189400	-0.00001100
N	-1.00648000	1.26941900	-0.00000700
C	-1.60111700	2.60522300	-0.00000700
H	-2.22022700	2.73920700	0.88430900
H	-0.79387500	3.32869100	-0.00003300
H	-2.22026600	2.73918700	-0.88429900
O	-3.06305100	0.30452900	0.00000900
C	-2.19140800	-2.22421600	0.00001100
H	-2.82446800	-2.18471600	-0.88346700
H	-1.60576100	-3.13509100	-0.00001800
H	-2.82442300	-2.18474700	0.88352300
C	3.32409300	-0.98813500	-0.00000100
H	3.24147900	-1.61432500	-0.88471400
H	4.28550600	-0.48225900	0.00001300
H	3.24146000	-1.61434100	0.88469800

DMSO

0 1

C	-2.44776500	1.35710100	0.00000100
H	-3.42857700	1.80266200	0.00000100
C	-0.90615400	-0.18318400	0.00000000
C	-0.12699000	-1.37545000	0.00000100
O	-0.55340300	-2.52708300	0.00000200
N	1.25762200	-1.09387500	0.00000000
C	1.84559400	0.17475200	-0.00000100
C	-0.35229300	1.07936700	0.00000000
N	-2.27629600	0.02243600	0.00000100
N	-1.30590000	2.04007700	0.00000000
N	1.00613600	1.26898900	-0.00000100
C	1.59890900	2.60591700	-0.00000100
H	2.21602000	2.74196500	-0.88528000
H	0.79097700	3.32825900	-0.00000300
H	2.21601700	2.74196700	0.88528000
O	3.06366700	0.30644300	-0.00000100
C	2.19366100	-2.22168100	0.00000000
H	2.82543100	-2.18297400	0.88433900
H	1.60982300	-3.13341200	-0.00000200
H	2.82543400	-2.18297200	-0.88433700
C	-3.32671500	-0.98814300	0.00000100
H	-3.24545800	-1.61266900	0.88587900
H	-4.28673200	-0.48049600	0.00000100
H	-3.24545900	-1.61266900	-0.88587800

H_2O

0 1

C	2.44783900	1.35693600	0.00000000
H	3.42866100	1.80246100	0.00000000
C	0.90616600	-0.18327100	0.00000000
C	0.12689900	-1.37538100	0.00000000
O	0.55313000	-2.52717600	0.00000000
N	-1.25769000	-1.09377600	0.00000000
C	-1.84550500	0.17482500	0.00000000
C	0.35231600	1.07930100	0.00000000
N	2.27631500	0.02234200	0.00000000
N	1.30593300	2.03997200	0.00000000
N	-1.00611300	1.26896900	0.00000000
C	-1.59875600	2.60596500	0.00000000
H	-2.21574600	2.74214800	0.88533800
H	-0.79076600	3.32822200	0.00000100
H	-2.21574500	2.74214900	-0.88533800
O	-3.06370400	0.30654900	0.00000100
C	-2.19378900	-2.22152800	-0.00000100
H	-2.82549500	-2.18284800	-0.88438100
H	-1.61005400	-3.13331000	-0.00000800
H	-2.82548500	-2.18285700	0.88438800
C	3.32683600	-0.98816100	0.00000000
H	3.24564500	-1.61259700	-0.88594200
H	4.28677800	-0.48041700	-0.00000300
H	3.24564900	-1.61259200	0.88594600

Appendix II. Cartesian cordinates of caffeine calculated at **BLYP/aug-cc-pVTZ** level of theory in vacuum and solvents in PCM model

Vacuum				$CHCl_3$			
0 1				0 1			
C	-2.46061200	1.38649500	-0.00000100	C	2.46383400	1.37805200	0.00000000
H	-3.44563700	1.83778000	-0.00000100	H	3.44938500	1.82728500	0.00000000
C	-0.91122000	-0.17874800	0.00000000	C	0.91224800	-0.18364600	0.00000000
C	-0.14340300	-1.39132300	0.00000000	C	0.13842700	-1.38924500	-0.00000100
O	-0.58777600	-2.54545400	0.00000000	O	0.57386600	-2.55046800	-0.00000100
N	1.26244400	-1.11161400	0.00000000	N	-1.26663000	-1.10742400	0.00000000
C	1.87257800	0.16794300	0.00000000	C	-1.86902300	0.17191400	0.00000100
C	-0.35071700	1.09409100	0.00000000	C	0.35189400	1.09052900	0.00000000
N	-2.29160900	0.03159700	0.00000000	N	2.29316800	0.02679400	0.00000000
N	-1.30953400	2.06913600	0.00000000	N	1.31120700	2.06383200	0.00000000
N	1.01737900	1.28000300	0.00000000	N	-1.01623900	1.27929100	0.00000000
C	1.62105200	2.62499700	0.00000000	C	-1.61551400	2.62767900	0.00000000
H	2.24772700	2.75386800	-0.88795100	H	-2.23816100	2.76062600	0.88968500
H	0.80909600	3.35318100	0.00000000	H	-0.80226400	3.35348700	0.00000100
H	2.24772600	2.75386800	0.88795300	H	-2.23816000	2.76062600	-0.88968500
O	3.09596400	0.29892800	0.00000000	O	-3.09737300	0.30553000	0.00000000
C	2.19663700	-2.25642500	0.00000000	C	-2.20410600	-2.25032800	0.00000100
H	2.83586600	-2.21724700	0.88721800	H	-2.84058500	-2.21318000	-0.88896800
H	1.59600300	-3.16604100	-0.00000200	H	-1.60795600	-3.16211100	0.00000100
H	2.83586800	-2.21724500	-0.88721700	H	-2.84058500	-2.21317900	0.88896900
C	-3.34382100	-0.99241300	0.00000100	C	3.35424500	-0.99071500	0.00000000
H	-3.25637400	-1.62560500	0.88756400	H	3.27281600	-1.62025500	-0.89012900
H	-4.31312200	-0.48629100	0.00000000	H	4.31819200	-0.47643000	0.00000000
H	-3.25637500	-1.62560600	-0.88756200	H	3.27281600	-1.62025500	0.89013000

DMSO				H_2O			
0 1				0 1			
C	-2.46515200	1.37499300	0.00000000	C	-2.46522500	1.37482000	-0.00000100
H	-3.45088000	1.82355800	0.00000100	H	-3.45096300	1.82335200	-0.00000100
C	-0.91258500	-0.18521200	-0.00000100	C	-0.91259900	-0.18529800	0.00000100
C	-0.13669400	-1.38793000	-0.00000300	C	-0.13659500	-1.38785700	0.00000100
O	-0.56883300	-2.55212500	-0.00000100	O	-0.56855600	-2.55221400	0.00000100
N	1.26787300	-1.10566800	0.00000000	N	1.26794200	-1.10556600	0.00000100
C	1.86721300	0.17324600	0.00000200	C	1.86711800	0.17332300	0.00000000
C	-0.35227900	1.08934600	0.00000000	C	-0.35230300	1.08928000	0.00000000
N	-2.29359200	0.02516500	0.00000000	N	-2.29361000	0.02506800	0.00000000
N	-1.31177100	2.06191500	0.00000000	N	-1.31180800	2.06180500	-0.00000100
N	1.01587900	1.27888700	0.00000000	N	1.01585600	1.27886800	0.00000100
C	1.61319900	2.62846100	0.00000100	C	1.61303800	2.62851400	0.00000100
H	2.23377300	2.76358500	-0.89066500	H	2.23348600	2.76378300	-0.89072400
H	0.79918500	3.35305800	-0.00000300	H	0.79896000	3.35302000	-0.00000300
H	2.23376700	2.76358700	0.89067000	H	2.23348100	2.76378600	0.89072900
O	3.09790700	0.30741400	0.00000000	O	3.09794100	0.30751400	-0.00000100
C	2.20650300	-2.24764200	0.00000000	C	2.20663500	-2.24748300	-0.00000100
H	2.84170200	-2.21125100	0.88980800	H	2.84176500	-2.21112800	0.88985200
H	1.61224900	-3.16036500	-0.00000300	H	1.61248500	-3.16026000	-0.00000300
H	2.84170600	-2.21124900	-0.88980500	H	2.84176500	-2.21112300	-0.88985400
C	-3.35685900	-0.99080600	0.00000000	C	-3.35697400	-0.99083100	0.00000000
H	-3.27674700	-1.61862700	0.89130900	H	-3.27692500	-1.61855700	0.89137400
H	-4.31939300	-0.47481100	0.00000100	H	-4.31943700	-0.47475200	-0.00000100
H	-3.27674800	-1.61862600	-0.89130900	H	-3.27692500	-1.61855600	-0.89137600

Appendix III. Cartesian cordinates of caffeine calculated at **BP86/aug-cc-pVTZ** level of theory in vacuum and solvents in PCM model

Vacuum

0 1

C	2.45159400	-1.38356800	0.00000000
H	3.44044300	-1.83404900	-0.00000100
C	0.90960900	0.17906200	0.00000100
C	0.14310200	1.38935200	0.00000000
O	0.58347700	2.54289700	0.00000000
N	-1.25481300	1.10612800	0.00000000
C	-1.86434600	-0.16684400	0.00000000
C	0.34911900	-1.09224700	0.00000000
N	2.28329700	-0.03254900	0.00000000
N	1.30293600	-2.06656900	0.00000000
N	-1.01422800	-1.27530200	0.00000000
C	-1.61601400	-2.61010300	0.00000000
H	-2.24471100	-2.73943900	-0.88978300
H	-0.80111400	-3.34011000	-0.00000300
H	-2.24470800	-2.73944100	0.88978400
O	-3.08618700	-0.29579200	0.00000000
C	-2.18353300	2.24206000	0.00000000
H	-2.82498800	2.20325200	0.88895700
H	-1.57813500	3.15289700	0.00000000
H	-2.82498900	2.20325200	-0.88895600
C	3.32743300	0.98698100	0.00000000
H	3.23871300	1.62226200	0.88927500
H	4.30033900	0.48215600	-0.00000100
H	3.23871100	1.62226400	-0.88927300

$CHCl_3$

0 1

C	2.45508200	-1.37498500	0.00000100
H	3.44452500	-1.82333900	0.00000100
C	0.91068600	0.18417000	0.00000000
C	0.13783000	1.38754600	0.00000000
O	0.56894400	2.54796300	0.00000000
N	-1.25941200	1.10196600	0.00000000
C	-1.86124800	-0.17113100	0.00000100
C	0.35045300	-1.08848400	0.00000000
N	2.28502100	-0.02730300	0.00000000
N	1.30493100	-2.06094100	0.00000000
N	-1.01288400	-1.27495300	0.00000000
C	-1.60972700	-2.61333500	0.00000000
H	-2.23418900	-2.74735200	-0.89159500
H	-0.79325400	-3.34069300	-0.00000500
H	-2.23418000	-2.74735500	0.89160000
O	-3.08762000	-0.30298500	0.00000000
C	-2.19147900	2.23592000	0.00000000
H	-2.83009000	2.19946700	0.89082000
H	-1.59065000	3.14901900	-0.00000200
H	-2.83009300	2.19946500	-0.89081900
C	3.33791300	0.98570300	0.00000000
H	3.25558200	1.61723900	0.89193900
H	4.30553000	0.47269200	0.00000200
H	3.25558500	1.61723600	-0.89194200

DMSO

0 1

C	2.45651900	-1.37187600	-0.00000100
H	3.44616100	-1.81953400	-0.00000100
C	0.91105300	0.18582300	0.00000000
C	0.13598100	1.38636000	0.00000100
O	0.56364000	2.54964200	0.00000200
N	-1.26082700	1.10023200	0.00000000
C	-1.85963900	-0.17260000	0.00000100
C	0.35090900	-1.08722100	-0.00000100
N	2.28552700	-0.02550700	0.00000000
N	1.30563000	-2.05889500	-0.00000100
N	-1.01243600	-1.27469900	0.00000000
C	-1.60715800	-2.61434400	0.00000000
H	-2.22946100	-2.75072500	-0.89261200
H	-0.78982200	-3.34039400	0.00000200
H	-2.22946300	-2.75072400	0.89261000
O	-3.08814100	-0.30513900	0.00000100
C	-2.19408500	2.23326600	0.00000000
H	-2.83138800	2.19764500	0.89168900
H	-1.59513700	3.14730900	-0.00000400
H	-2.83139200	2.19764000	-0.89168600
C	3.34059100	0.98595400	0.00000000
H	3.25971500	1.61573500	0.89315400
H	4.30679800	0.47119600	0.00000000
H	3.25971500	1.61573600	-0.89315300

H_2O

0 1

C	2.45659900	-1.37169900	0.00000000
H	3.44625200	-1.81932200	0.00000000
C	0.91106900	0.18591500	0.00000000
C	0.13587600	1.38629600	0.00000000
O	0.56334500	2.54973300	0.00000000
N	-1.26090600	1.10013200	0.00000000
C	-1.85955400	-0.17268500	0.00000000
C	0.35093700	-1.08715100	0.00000000
N	2.28554900	-0.02540000	0.00000000
N	1.30567500	-2.05877800	0.00000000
N	-1.01240700	-1.27468800	0.00000000
C	-1.60698100	-2.61441000	0.00000000
H	-2.22915600	-2.75094600	-0.89267000
H	-0.78957400	-3.34036300	-0.00000100
H	-2.22915500	-2.75094700	0.89267100
O	-3.08817300	-0.30525700	0.00000000
C	-2.19423200	2.23310700	0.00000000
H	-2.83146400	2.19751900	0.89173500
H	-1.59539000	3.14720600	0.00000000
H	-2.83146400	2.19752000	-0.89173500
C	3.34071100	0.98599000	0.00000000
H	3.25990400	1.61567500	0.89322100
H	4.30684600	0.47114400	0.00000000
H	3.25990500	1.61567500	-0.89322100

Appendix IV. Cartesian cordinates of caffeine calculated at **CAM-B3LYP/aug-cc-pVTZ** level of theory in vacuum and solvents in PCM model

Vacuum				*$CHCl_3$*			
0 1				0 1			
C	2.43602000	1.35947500	0.00000000	C	2.43935700	1.35151000	0.00000100
H	3.41654300	1.80640400	0.00000100	H	3.42051600	1.79642300	0.00000300
C	0.90184600	-0.17356000	-0.00000200	C	0.90275100	-0.17846600	0.00000000
C	0.12776800	-1.37217900	-0.00000100	C	0.12316700	-1.37003000	-0.00000200
O	0.56573600	-2.50758600	0.00000000	O	0.55195400	-2.51251500	-0.00000100
N	-1.24701800	-1.09465000	-0.00000200	N	-1.25114700	-1.09045200	-0.00000200
C	-1.83948700	0.17100500	0.00000100	C	-1.83638200	0.17474300	-0.00000100
C	0.35159400	1.07871700	-0.00000200	C	0.35277000	1.07514900	0.00000000
N	2.26623100	0.02806200	0.00000000	N	2.26784800	0.02357400	0.00000100
N	1.30239600	2.03659900	-0.00000200	N	1.30377400	2.03154800	0.00000100
N	-1.00383300	1.26564000	-0.00000300	N	-1.00276900	1.26486200	-0.00000100
C	-1.60115600	2.59134000	-0.00000100	C	-1.59541300	2.59391900	0.00000100
H	-2.22393100	2.72113700	0.88157500	H	-2.21411300	2.72825200	0.88336200
H	-0.79618200	3.31735900	-0.00003000	H	-0.78902900	3.31744700	-0.00001300
H	-2.22397800	2.72111600	-0.88154700	H	-2.21413700	2.72824400	-0.88334300
O	-3.04568800	0.29614000	0.00000500	O	-3.04745700	0.30277200	0.00000200
C	-2.17785100	-2.21734800	0.00000000	C	-2.18476100	-2.21161600	-0.00000200
H	-2.81310100	-2.17375200	-0.88059600	H	-2.81720500	-2.17041800	-0.88248000
H	-1.59099000	-3.12735900	-0.00002400	H	-1.60215100	-3.12353300	-0.00001300
H	-2.81306800	-2.17378000	0.88062300	H	-2.81719000	-2.17043200	0.88248900
C	3.29806700	-0.98995400	0.00000300	C	3.30804400	-0.98850700	0.00000100
H	3.20658900	-1.61878600	-0.88111900	H	3.22255500	-1.61373300	-0.88378000
H	4.26591300	-0.49650800	0.00000600	H	4.27109300	-0.48749400	0.00000900
H	3.20658400	-1.61878600	0.88112600	H	3.22254400	-1.61374400	0.88377300

DMSO				*H_2O*			
0 1				0 1			
C	-2.44072400	1.34862800	-0.00000100	C	-2.44080000	1.34846500	-0.00000100
H	-3.42213100	1.79282000	-0.00000200	H	-3.42222000	1.79262000	-0.00000100
C	-0.90302400	-0.18004400	-0.00000100	C	-0.90303400	-0.18013000	-0.00000200
C	-0.12160500	-1.36874100	-0.00000100	C	-0.12151700	-1.36867200	0.00000000
O	-0.54703800	-2.51415400	-0.00000200	O	-0.54676800	-2.51424300	0.00000400
N	1.25238800	-1.08874800	0.00000000	N	1.25245600	-1.08865100	-0.00000200
C	1.83476600	0.17596400	0.00000200	C	1.83467900	0.17603400	0.00000100
C	-0.35315100	1.07397000	0.00000000	C	-0.35317300	1.07390500	-0.00000100
N	-2.26829600	0.02207000	-0.00000100	N	-2.26831500	0.02198000	-0.00000100
N	-1.30425400	2.02976300	-0.00000100	N	-1.30428600	2.02966200	-0.00000100
N	1.00242400	1.26442200	0.00000100	N	1.00240200	1.26440000	-0.00000100
C	1.59322700	2.59456000	0.00000400	C	1.59308300	2.59460100	0.00000200
H	2.20991000	2.73098800	-0.88434400	H	2.20964600	2.73116100	-0.88440400
H	0.78619900	3.31700300	-0.00000100	H	0.78600200	3.31696600	-0.00000300
H	2.20990100	2.73098900	0.88435700	H	2.20963700	2.73116200	0.88441400
O	3.04809600	0.30479200	0.00000400	O	3.04813200	0.30490200	0.00000400
C	2.18696000	-2.20916000	-0.00000100	C	2.18708200	-2.20901500	-0.00000300
H	2.81808800	-2.16882900	0.88336900	H	2.81812100	-2.16873500	0.88342900
H	1.60608700	-3.12186500	-0.00000900	H	1.60630700	-3.12176500	-0.00003600
H	2.81809700	-2.16881900	-0.88336500	H	2.81816200	-2.16869500	-0.88340200
C	-3.31061900	-0.98852400	-0.00000200	C	-3.31073700	-0.98853900	0.00000000
H	-3.22656200	-1.61207000	0.88498000	H	-3.22675500	-1.61198700	0.88505300
H	-4.27231400	-0.48572100	-0.00000100	H	-4.27235900	-0.48563700	-0.00000500
H	-3.22656300	-1.61206800	-0.88498600	H	-3.22674900	-1.61199500	-0.88504700

Appendix V. Cartesian cordinates of caffeine calculated at **LC-BLYP/aug-cc-pVTZ** level of theory in vacuum and solvents in PCM model

Vacuum

0 1

C	-2.42511400	1.35002500	-0.00000200
H	-3.40689400	1.79645700	-0.00000300
C	-0.89766000	-0.16851600	-0.00000100
C	-0.12303500	-1.36202200	-0.00000100
O	-0.56210200	-2.48987700	-0.00000200
N	1.23942400	-1.08869600	-0.00000200
C	1.82601000	0.17094200	0.00000000
C	-0.35126100	1.07219700	-0.00000200
N	-2.25465900	0.02909600	0.00000000
N	-1.29833700	2.02252200	-0.00000300
N	0.99965000	1.25909000	-0.00000100
C	1.59469900	2.57469600	0.00000200
H	2.21795800	2.70503100	-0.88038500
H	0.79129400	3.30231800	0.00000800
H	2.21796500	2.70502200	0.88038500
O	3.02683600	0.29200500	0.00000400
C	2.16556500	-2.20209700	-0.00000300
H	2.80124700	-2.15817000	0.87933200
H	1.58111400	-3.11344500	-0.00001500
H	2.80126200	-2.15815500	-0.87932600
C	-3.27543900	-0.98771200	0.00000700
H	-3.18035500	-1.61681900	0.87978400
H	-4.24626800	-0.50156600	-0.00001800
H	-3.18032900	-1.61685600	-0.87974100

$CHCl_3$

0 1

C	2.43761700	1.34686300	0.00000000
H	3.42662200	1.79405200	0.00000100
C	0.90285500	-0.17534200	0.00000000
C	0.11834600	-1.36462000	-0.00000100
O	0.54857000	-2.50642400	0.00000000
N	-1.24737800	-1.08769200	0.00000000
C	-1.82679700	0.17539600	0.00000000
C	0.35406500	1.07342200	0.00000000
N	2.26377500	0.02398300	0.00000000
N	1.30404600	2.02721500	0.00000000
N	-1.00161800	1.26293600	0.00000000
C	-1.59342200	2.58409600	0.00000000
H	-2.21649500	2.71807600	0.88917600
H	-0.78183700	3.31261300	-0.00000200
H	-2.21649900	2.71807500	-0.88917300
O	-3.03982100	0.30008000	0.00000100
C	-2.17883100	-2.20080700	0.00000000
H	-2.81554500	-2.15637200	-0.88802400
H	-1.59374400	-3.11965200	-0.00000200
H	-2.81554100	-2.15637400	0.88802700
C	3.29336100	-0.99097600	0.00000000
H	3.20095500	-1.61943500	-0.88961500
H	4.26618500	-0.49408800	0.00000100
H	3.20095500	-1.61943600	0.88961600

DMSO

0 1

C	2.43031100	1.33884600	0.00000100
H	3.41339900	1.78201400	0.00000100
C	0.89871600	-0.17528000	0.00000000
C	0.11657600	-1.35880500	0.00000000
O	0.54259600	-2.49666400	0.00000400
N	-1.24511700	-1.08259900	0.00000000
C	-1.82142900	0.17614300	-0.00000100
C	0.35313900	1.06725800	0.00000000
N	2.25675000	0.02279300	0.00000000
N	1.30050500	2.01555500	0.00000100
N	-0.99796700	1.25797600	-0.00000100
C	-1.58603200	2.57801900	0.00000000
H	-2.20296600	2.71562200	0.88332000
H	-0.78049100	3.30196400	-0.00000100
H	-2.20296700	2.71562300	-0.88332000
O	-3.02930200	0.30149800	-0.00000300
C	-2.17508800	-2.19360900	0.00000100
H	-2.80650100	-2.15304200	-0.88227300
H	-1.59686500	-3.10771500	-0.00000600
H	-2.80649100	-2.15304900	0.88228300
C	3.28768900	-0.98663500	-0.00000100
H	3.20065900	-1.61033100	-0.88387900
H	4.25271400	-0.49111300	0.00000500
H	3.20065300	-1.61033800	0.88387200

H_2O

0 1

C	2.43920000	1.34378100	0.00000000
H	3.42862700	1.79002100	0.00000000
C	0.90310000	-0.17703400	0.00000000
C	0.11664200	-1.36333700	0.00000000
O	0.54316700	-2.50817300	0.00000000
N	-1.24877500	-1.08588500	0.00000000
C	-1.82519300	0.17674800	0.00000000
C	0.35453900	1.07214500	0.00000000
N	2.26425000	0.02232600	0.00000000
N	1.30466900	2.02531200	0.00000000
N	-1.00116700	1.26254100	0.00000000
C	-1.59091800	2.58484100	0.00000000
H	-2.21167900	2.72125900	0.89032900
H	-0.77866700	3.31221000	-0.00000200
H	-2.21168300	2.72125800	-0.89032600
O	-3.04051600	0.30249700	0.00000100
C	-2.18118500	-2.19825600	0.00000000
H	-2.81637200	-2.15491100	-0.88907600
H	-1.59794700	-3.11791000	-0.00000300
H	-2.81636900	-2.15491400	0.88907800
C	3.29587300	-0.99117900	0.00000000
H	3.20514100	-1.61771600	-0.89101700
H	4.26740100	-0.49248700	0.00000000
H	3.20514000	-1.61771600	0.89101800

Appendix VI. Cartesian cordinates of caffeine calculated at **M06/aug-cc-pVTZ** level of theory in vacuum and solvents in PCM model

Vacuum

0 1

C	-2.42693300	1.36795000	0.00000000
H	-3.41182200	1.81134600	0.00000000
C	-0.90098000	-0.17265800	0.00000000
C	-0.13958800	-1.37585700	0.00000000
O	-0.57874100	-2.50732800	0.00000000
N	1.24167400	-1.09620200	0.00000000
C	1.84604900	0.16541200	0.00000000
C	-0.34845000	1.08091300	0.00000000
N	-2.26371400	0.03300900	0.00000000
N	-1.29316000	2.04355500	0.00000000
N	1.00594600	1.26294100	0.00000000
C	1.60412900	2.58472600	0.00000000
H	2.22913800	2.71582100	-0.88190600
H	0.80005200	3.31465200	-0.00000600
H	2.22912900	2.71582600	0.88191200
O	3.04827700	0.28901000	0.00000000
C	2.16637600	-2.21959000	0.00000000
H	2.80439600	-2.18072800	0.88103200
H	1.57680100	-3.13035500	0.00000100
H	2.80439500	-2.18073000	-0.88103200
C	-3.29310700	-0.98182800	0.00000000
H	-3.20273000	-1.61391400	0.88127900
H	-4.26311900	-0.48899000	0.00000200
H	-3.20273300	-1.61391100	-0.88128200

$CHCl_3$

0 1

C	-1.80274600	2.12318100	0.00000000
H	-2.42627200	3.00456300	0.00000000
C	0.00000000	0.91919700	0.00000000
C	1.32182000	0.39713000	0.00000000
O	2.35574900	1.03871300	0.00000000
N	1.31137600	-1.01198800	0.00000000
C	0.18859700	-1.84054900	0.00000000
C	-1.12501200	0.13577700	0.00000000
N	-0.46428600	2.21729700	0.00000000
N	-2.24994100	0.87808200	0.00000000
N	-1.04545500	-1.22898900	0.00000000
C	-2.23114400	-2.06719000	0.00000000
H	-2.24449900	-2.70267200	0.88372100
H	-3.10053100	-1.41726400	0.00000000
H	-2.24449900	-2.70267200	-0.88372100
O	0.29756000	-3.04900100	0.00000000
C	2.59067400	-1.70606100	0.00000000
H	2.67684400	-2.33670000	-0.88270600
H	3.37344400	-0.95570700	0.00000000
H	2.67684400	-2.33670000	0.88270600
C	0.32825800	3.42837500	0.00000000
H	0.96174700	3.46407800	-0.88358500
H	-0.34583900	4.28132700	0.00000000
H	0.96174700	3.46407800	0.88358500

DMSO

0 1

C	2.43130000	1.35835400	0.00000200
H	3.41632100	1.79996400	0.00000300
C	0.90223300	-0.17792800	0.00000000
C	0.13435500	-1.37203700	-0.00000200
O	0.56278400	-2.51249700	-0.00000500
N	-1.24625000	-1.09112400	-0.00000100
C	-1.84103000	0.16962200	0.00000000
C	0.34963100	1.07688700	0.00000200
N	2.26543800	0.02813100	0.00000000
N	1.29447000	2.03759000	0.00000300
N	-1.00512200	1.26167200	0.00000100
C	-1.59813400	2.58729100	0.00000200
H	-2.21726000	2.72437900	0.88462800
H	-0.79269000	3.31453900	-0.00000600
H	-2.21727300	2.72437400	-0.88461500
O	-3.04997300	0.29583800	-0.00000100
C	-2.17411700	-2.21257100	-0.00000100
H	-2.80817800	-2.17729000	-0.88371300
H	-1.59018300	-3.12592800	0.00000900
H	-2.80819100	-2.17727800	0.88370100
C	3.30387800	-0.98082900	0.00000000
H	3.21889500	-1.60765800	-0.88486500
H	4.26872500	-0.48078700	-0.00000500
H	3.21890100	-1.60765400	0.88486800

H_2O

0 1

C	-2.43137500	1.35820100	0.00000000
H	-3.41639900	1.79978300	0.00000000
C	-0.90224600	-0.17800400	0.00000000
C	-0.13427100	-1.37196600	0.00000000
O	-0.56253400	-2.51257200	-0.00000100
N	1.24631400	-1.09103500	0.00000000
C	1.84093900	0.16968700	0.00000000
C	-0.34965200	1.07682700	0.00000000
N	-2.26545400	0.02804900	0.00000000
N	-1.29450000	2.03749500	0.00000000
N	1.00510300	1.26165100	0.00000000
C	1.59800600	2.58732600	0.00000000
H	2.21702600	2.72453700	-0.88467700
H	0.79251600	3.31450700	-0.00000600
H	2.21701700	2.72454200	0.88468100
O	3.04999800	0.29593600	0.00000100
C	2.17423300	-2.21243400	0.00000000
H	2.80823600	-2.17718000	0.88375000
H	1.59039800	-3.12583900	0.00000700
H	2.80822600	-2.17718900	-0.88375800
C	-3.30398200	-0.98085200	0.00000100
H	-3.21905900	-1.60758500	0.88493400
H	-4.26876600	-0.48073500	-0.00000300
H	-3.21905500	-1.60759100	-0.88492900

Appendix VII. Cartesian cordinates of caffeine calculated at **PBE0/aug-cc-pVTZ** level of theory in vacuum and solvents in PCM model

Vacuum

0 1

C	2.43232800	1.36183100	-0.00000900
H	3.41531100	1.80852800	-0.00001200
C	0.90282000	-0.17717000	-0.00000200
C	0.13127700	-1.37614400	0.00000200
O	0.56504100	-2.51527400	0.00000700
N	-1.24410700	-1.09289000	0.00000500
C	-1.83957700	0.16930400	0.00000500
C	0.34987300	1.08066800	-0.00000500
N	2.26422700	0.02847000	-0.00000400
N	1.29762000	2.04144500	-0.00001000
N	-1.00260600	1.26465600	-0.00000200
C	-1.59957800	2.58556400	-0.00000400
H	-2.22425100	2.71546800	0.88331300
H	-0.79300800	3.31369600	-0.00002000
H	-2.22427500	2.71545400	-0.88330600
O	-3.04711700	0.29572600	0.00000700
C	-2.17202100	-2.21165000	0.00001100
H	-2.80941300	-2.16880600	-0.88230600
H	-1.58184900	-3.12293000	0.00001100
H	-2.80940600	-2.16880200	0.88233400
C	3.29522200	-0.98402100	-0.00000300
H	3.20569200	-1.61525800	-0.88283300
H	4.26411400	-0.48775800	0.00000200
H	3.20568400	-1.61526400	0.88282100

$CHCl_3$

0 1

C	2.43569500	1.35385100	-0.00000900
H	3.41927600	1.79860200	-0.00001200
C	0.90383600	-0.18215600	-0.00000200
C	0.12647400	-1.37424800	0.00000400
O	0.55100400	-2.52022900	0.00001000
N	-1.24844400	-1.08880300	0.00000500
C	-1.83681800	0.17331400	0.00000200
C	0.35111700	1.07699100	-0.00000500
N	2.26594400	0.02363300	-0.00000400
N	1.29922600	2.03615200	-0.00001000
N	-1.00139900	1.26419600	-0.00000400
C	-1.59330400	2.58864500	-0.00000500
H	-2.21377100	2.72341200	0.88513400
H	-0.78502800	3.31401500	-0.00002200
H	-2.21379500	2.72339700	-0.88512900
O	-3.04882700	0.30255400	0.00000700
C	-2.17930500	-2.20584300	0.00001000
H	-2.81392300	-2.16542000	-0.88417800
H	-1.59352200	-3.11918200	-0.00000500
H	-2.81389700	-2.16543500	0.88421900
C	3.30518400	-0.98290700	-0.00000300
H	3.22171600	-1.61050700	-0.88547700
H	4.26926500	-0.47908800	0.00000100
H	3.22171100	-1.61051000	0.88546800

DMSO

0 1

C	-2.43707700	1.35095300	0.00000000
H	-3.42088800	1.79499800	-0.00000100
C	-0.90415900	-0.18378400	0.00000100
C	-0.12481500	-1.37307800	0.00000300
O	-0.54595400	-2.52188100	0.00000100
N	1.24979800	-1.08715100	0.00000100
C	1.83537200	0.17466400	0.00000100
C	-0.35153800	1.07576100	0.00000000
N	-2.26644100	0.02197700	0.00000100
N	-1.29979800	2.03424900	-0.00000100
N	1.00098800	1.26389100	0.00000000
C	1.59090300	2.58951300	-0.00000200
H	2.20932100	2.72648100	-0.88612900
H	0.78186000	3.31369000	-0.00000500
H	2.20931700	2.72648500	0.88612800
O	3.04943300	0.30470900	-0.00000200
C	2.18168700	-2.20339900	-0.00000100
H	2.81501000	-2.16387100	0.88507000
H	1.59767700	-3.11757900	-0.00000500
H	2.81501200	-2.16386500	-0.88507100
C	-3.30782700	-0.98304000	0.00000000
H	-3.22586200	-1.60895600	0.88667100
H	-4.27051200	-0.47736400	0.00000000
H	-3.22586200	-1.60895700	-0.88667000

H_2O

0 1

C	2.43715300	1.35079000	0.00000000
H	3.42097600	1.79480000	0.00000000
C	0.90417200	-0.18387200	0.00000000
C	0.12472200	-1.37301500	0.00000000
O	0.54567900	-2.52196900	0.00000000
N	-1.24987100	-1.08705800	0.00000000
C	-1.83529400	0.17474000	0.00000000
C	0.35156300	1.07569300	0.00000000
N	2.26646300	0.02188100	0.00000000
N	1.29983400	2.03414200	0.00000000
N	-1.00096200	1.26387600	0.00000000
C	-1.59075100	2.58956500	0.00000000
H	-2.20904500	2.72667200	0.88618800
H	-0.78165100	3.31366100	-0.00000100
H	-2.20904800	2.72667100	-0.88618500
O	-3.04946700	0.30482500	0.00000000
C	-2.18181800	-2.20325600	0.00000000
H	-2.81507300	-2.16376000	-0.88511700
H	-1.59790800	-3.11748400	-0.00000400
H	-2.81506900	-2.16376500	0.88512000
C	3.30795100	-0.98305900	0.00000000
H	3.22606200	-1.60888400	-0.88673700
H	4.27055900	-0.47728000	0.00000000
H	3.22606200	-1.60888400	0.88673700

Appendix VIII. Cartesian cordinates of caffeine calculated at **TPSSh/aug-cc-pVTZ** level of theory in vacuum and solvents in PCM model

Vacuum

0 1

C	-2.43987400	1.37333800	0.00000000
H	-3.42101000	1.82268400	0.00000100
C	-0.90530300	-0.17829300	-0.00000200
C	-0.13818400	-1.38233400	-0.00000100
O	-0.57193900	-2.52980500	0.00000100
N	1.24752500	-1.09830800	-0.00000100
C	1.84978800	0.16741600	0.00000000
C	-0.34965100	1.08411200	-0.00000100
N	-2.27461100	0.03160700	-0.00000100
N	-1.29866500	2.05395100	0.00000000
N	1.00839700	1.26887200	-0.00000100
C	1.61235300	2.60052400	0.00000100
H	2.23555000	2.72331000	-0.88525500
H	0.80244700	3.32498700	0.00000100
H	2.23555000	2.72330800	0.88525700
O	3.06470300	0.29363600	0.00000100
C	2.18147800	-2.22838200	0.00000000
H	2.81573000	-2.18148700	0.88437700
H	1.58354400	-3.13477400	0.00000000
H	2.81573100	-2.18148700	-0.88437600
C	-3.31878300	-0.98541600	0.00000100
H	-3.22824900	-1.61336300	0.88493000
H	-4.28262100	-0.47909900	-0.00000100
H	-3.22824800	-1.61336700	-0.88492700

CHCl$_3$

0 1

C	-2.44328100	1.36496600	0.00000100
H	-3.42491400	1.81243500	0.00000200
C	-0.90641300	-0.18342200	-0.00000100
C	-0.13313300	-1.38046300	-0.00000200
O	-0.55733000	-2.53497200	0.00000100
N	1.25201800	-1.09412500	-0.00000100
C	1.84681100	0.17161300	0.00000000
C	-0.35095500	1.08036800	-0.00000100
N	-2.27633600	0.02656500	0.00000000
N	-1.30037700	2.04843300	0.00000000
N	1.00710700	1.26842700	-0.00000100
C	1.60595200	2.60384600	0.00000000
H	2.22512500	2.73116100	-0.88698300
H	0.79419000	3.32533000	-0.00000800
H	2.22511100	2.73116700	0.88699200
O	3.06633900	0.30067300	0.00000200
C	2.18913600	-2.22236900	0.00000000
H	2.82068400	-2.17759800	0.88615000
H	1.59552800	-3.13083100	-0.00000600
H	2.82069300	-2.17759100	-0.88614400
C	-3.32944800	-0.98416400	0.00000000
H	-3.24470800	-1.60861500	0.88742000
H	-4.28796500	-0.46980800	0.00000100
H	-3.24470900	-1.60861400	-0.88742100

DMSO

0 1

C	-2.44467200	1.36194600	0.00000000
H	-3.42647400	1.80877000	0.00000000
C	-0.90678400	-0.18508100	-0.00000100
C	-0.13140100	-1.37927100	-0.00000100
O	-0.55208200	-2.53668500	0.00000000
N	1.25339400	-1.09242100	-0.00000100
C	1.84525000	0.17302100	0.00000000
C	-0.35139400	1.07912600	0.00000000
N	-2.27683300	0.02485700	0.00000000
N	-1.30096500	2.04645200	0.00000000
N	1.00668000	1.26812300	0.00000000
C	1.60344900	2.60481100	0.00000100
H	2.22063300	2.73428600	-0.88793300
H	0.79081800	3.32496800	-0.00000700
H	2.22061900	2.73429200	0.88794500
O	3.06692200	0.30280000	0.00000100
C	2.19163200	-2.21978600	-0.00000100
H	2.82195200	-2.17574900	0.88696400
H	1.59982600	-3.12912900	-0.00000800
H	2.82195900	-2.17574100	-0.88696100
C	-3.33225500	-0.98431800	0.00000100
H	-3.24883600	-1.60714000	0.88855300
H	-4.28927300	-0.46809700	-0.00000500
H	-3.24882900	-1.60714900	-0.88854500

H$_2$O

0 1

C	-2.44475000	1.36177500	0.00000000
H	-3.42656100	1.80856600	0.00000000
C	-0.90680000	-0.18517300	0.00000000
C	-0.13130200	-1.37920600	0.00000100
O	-0.55179100	-2.53677800	0.00000200
N	1.25347000	-1.09232300	0.00000000
C	1.84516700	0.17310200	0.00000000
C	-0.35142000	1.07905600	0.00000000
N	-2.27685500	0.02475400	0.00000000
N	-1.30100400	2.04633900	0.00000000
N	1.00665200	1.26810900	0.00000000
C	1.60328200	2.60487300	0.00000100
H	2.22034600	2.73448700	-0.88799100
H	0.79058300	3.32493500	-0.00000600
H	2.22033600	2.73449200	0.88799900
O	3.06695700	0.30291600	0.00000000
C	2.19177200	-2.21963200	-0.00000100
H	2.82202600	-2.17562200	0.88700700
H	1.60006900	-3.12902700	-0.00000400
H	2.82203000	-2.17561700	-0.88700600
C	-3.33238500	-0.98434300	-0.00000100
H	-3.24902900	-1.60708100	0.88861100
H	-4.28932400	-0.46802300	-0.00000100
H	-3.24902800	-1.60708200	-0.88861300

Appendix IX. Cartesian cordinates of caffeine calculated at **wB97X/aug-cc-pVTZ** level of theory in vacuum and solvents in PCM model

Vacuum

0 1

C	2.43791600	1.35934600	-0.00065400
H	3.42131300	1.80458300	-0.00113200
C	0.90310800	-0.17191000	0.00028700
C	0.12706700	-1.37490300	-0.00004100
O	0.56764800	-2.50983100	-0.00050000
N	-1.24739800	-1.09641600	0.00008100
C	-1.84022700	0.17198700	0.00017900
C	0.35429500	1.08014300	0.00014600
N	2.26738400	0.02853100	-0.00038600
N	1.30503100	2.04118000	-0.00041600
N	-1.00410200	1.26725600	0.00022200
C	-1.60120700	2.59289100	0.00017100
H	-2.22429900	2.72242700	0.88304500
H	-0.79462200	3.31990900	0.00037000
H	-2.22394500	2.72255600	-0.88293600
O	-3.04749900	0.29668200	0.00017400
C	-2.18033400	-2.21750200	-0.00025000
H	-2.81527800	-2.17214600	-0.88243700
H	-1.59513900	-3.13057700	-0.00009500
H	-2.81585100	-2.17215300	0.88151600
C	3.29536500	-0.99416400	0.00088300
H	3.19288200	-1.62890500	-0.87617900
H	4.26647400	-0.50412400	-0.00925500
H	3.20496500	-1.61556100	0.88886400

$CHCl_3$

0 1

C	-2.44142400	1.35146400	-0.00000400
H	-3.42537200	1.79468200	-0.00000600
C	-0.90402100	-0.17677200	0.00000000
C	-0.12243000	-1.37276000	0.00000100
O	-0.55395200	-2.51453600	0.00000200
N	1.25161900	-1.09232100	0.00000200
C	1.83720300	0.17565400	0.00000200
C	-0.35544700	1.07654400	-0.00000100
N	-2.26906100	0.02403800	-0.00000200
N	-1.30643500	2.03603700	-0.00000400
N	1.00309200	1.26654400	-0.00000200
C	1.59573000	2.59546400	0.00000000
H	2.21461100	2.72955800	-0.88476900
H	0.78791500	3.32021300	-0.00003700
H	2.21455400	2.72958500	0.88480500
O	3.04919200	0.30336300	0.00000300
C	2.18729300	-2.21198900	0.00000300
H	2.81970700	-2.16909300	0.88388400
H	1.60629700	-3.12695300	0.00001400
H	2.81969400	-2.16910700	-0.88388800
C	-3.30548300	-0.99268400	-0.00000100
H	-3.21533400	-1.61714200	0.88530800
H	-4.27170600	-0.49481400	-0.00001500
H	-3.21531700	-1.61716000	-0.88529600

DMSO

0 1

C	-2.44285600	1.34868600	-0.00000500
H	-3.42700200	1.79122300	-0.00000800
C	-0.90430300	-0.17827400	-0.00001200
C	-0.12090700	-1.37144100	-0.00002200
O	-0.54917100	-2.51602000	-0.00004200
N	1.25285800	-1.09068200	-0.00000400
C	1.83562800	0.17680800	0.00002200
C	-0.35579200	1.07538300	0.00001200
N	-2.26954200	0.02260200	-0.00002200
N	-1.30689600	2.03426300	0.00001700
N	1.00280800	1.26612300	0.00002800
C	1.59371500	2.59609700	0.00005300
H	2.21055200	2.73231000	-0.88572500
H	0.78533600	3.31985900	0.00004900
H	2.21052300	2.73229000	0.88585500
O	3.04980700	0.30532100	0.00003700
C	2.18942000	-2.20970800	-0.00001000
H	2.82049400	-2.16775600	0.88478600
H	1.61010600	-3.12543000	-0.00003900
H	2.82052500	-2.16771800	-0.88478200
C	-3.30802800	-0.99273400	-0.00004900
H	-3.21927600	-1.61554100	0.88645300
H	-4.27293200	-0.49317700	-0.00004200
H	-3.21926800	-1.61550200	-0.88657700

H_2O

0 1

C	-2.44293300	1.34853400	-0.00000700
H	-3.42708900	1.79103700	-0.00000800
C	-0.90431400	-0.17835300	-0.00001400
C	-0.12082500	-1.37136900	-0.00002300
O	-0.54891800	-2.51609900	-0.00004500
N	1.25292200	-1.09059000	-0.00000400
C	1.83554500	0.17687100	0.00002300
C	-0.35581100	1.07532100	0.00000900
N	-2.26956300	0.02252100	-0.00002300
N	-1.30692400	2.03416500	0.00001300
N	1.00279100	1.26610100	0.00002600
C	1.59358500	2.59613600	0.00005500
H	2.21030500	2.73248100	-0.88577900
H	0.78516000	3.31982700	0.00004900
H	2.21027100	2.73245700	0.88591800
O	3.04984300	0.30542100	0.00004300
C	2.18953200	-2.20957700	-0.00000800
H	2.82052900	-2.16766800	0.88484000
H	1.61030900	-3.12534000	-0.00004200
H	2.82056700	-2.16762800	-0.88482700
C	-3.30814000	-0.99275000	-0.00004500
H	-3.21944400	-1.61546700	0.88652100
H	-4.27297600	-0.49310800	-0.00002900
H	-3.21945100	-1.61542500	-0.88664200

References

1. Sitkowski, J.; Stefaniak, L.; Nicol, L.; Martin, M. L.; Martin, G. J.; Webb, G. A., Complete assignments of the 1H, 13C and 15N NMR spectra of caffeine. *Spectrochimica Acta Part A: Molecular and Biomolecular Spectroscopy* **1995,** 51, (5), 839-841.
